# Biodistribution of degradable polyanhydride particles in *Aedes aegypti* tissues

**DOI:** 10.1371/journal.pntd.0008365

**Published:** 2020-09-08

**Authors:** Edmund J. Norris, Adam S. Mullis, Yashdeep Phanse, Balaji Narasimhan, Joel R. Coats, Lyric C. Bartholomay

**Affiliations:** 1 Department of Entomology, Iowa State University, Ames, Iowa, United States of America; 2 Department of Chemical and Biological Engineering, Iowa State University, Ames, Iowa, United States of America; 3 Department of Pathobiological Sciences, University of Wisconsin, Madison, Wisconsin, United States of America; 4 Nanovaccine Institute, Iowa State University, Ames, Iowa, United States of America; International Centre for Genetic Engineering and Biotechnology, INDIA

## Abstract

Insecticide resistance poses a significant threat to the control of arthropods that transmit disease agents. Nanoparticle carriers offer exciting opportunities to expand the armamentarium of insecticides available for public health and other pests. Most chemical insecticides are delivered by contact or feeding, and from there must penetrate various biological membranes to reach target organs and kill the pest organism. Nanoparticles have been shown to improve bioactive compound navigation of such barriers in vertebrates, but have not been well-explored in arthropods. In this study, we explored the potential of polyanhydride micro- and nanoparticles (250 nm– 3 μm), labeled with rhodamine B to associate with and/or transit across insect biological barriers, including the cuticle, epithelium, midgut and ovaries, in female *Ae*. *aeygpti* mosquitoes. Mosquitoes were exposed using conditions to mimic surface contact with a residual spray or paint, topical exposure to mimic contact with aerosolized insecticide, or *per os* in a sugar meal. In surface contact experiments, microparticles were sometimes observed in association with the exterior of the insect cuticle. Nanoparticles were more uniformly distributed across exterior tissues and present at higher concentrations. Furthermore, by surface contact, topical exposure, or *per os*, particles were detected in internal organs. In every experiment, amphiphilic polyanhydride nanoparticles associated with internal tissues to a higher degree than hydrophobic nanoparticles. In vitro, nanoparticles associated with *Aedes aegypti* Aag2 cells within two hours of exposure, and particles were evident in the cytoplasm. Further studies demonstrated that particle uptake is dependent on caveolae-mediated endocytosis. The propensity of these nanoparticles to cross biological barriers including the cuticle, to localize in target tissue sites of interest, and to reach the cytoplasm of cells, provides great promise for targeted delivery of insecticidal candidates that cannot otherwise reach these cellular and subcellular locations.

## Author summary

To expand the repertoire of insecticides available for public health purposes, we explored the potential of biodegradable polyanhydride nanoparticles for localizing within specific tissues within the yellow fever mosquito, *Aedes aegypti*, after a variety of exposures. These nanoparticles successfully migrated across the insect cuticle, one of the primary barriers to insecticide potency, and localized within specific tissues observed in this study. We also demonstrated that these nanoparticles associated with an insect cell line and were internalized within these cells. In all exposures, these nanoparticles did not produce toxicity to the exposed organisms, making them ideal carriers for future insecticides. This study demonstrates the utility of these biodegradable nanoparticles in the future of insecticide development.

## Introduction

The uptake and biodistribution of an insecticidal active ingredient in target arthropod species is fundamental to its efficacy and to formulation of end use products. Successful insecticides have to migrate through the cuticle, distribute among various physiologically-relevant tissues within the pest, and reach target sites in high enough quantities to cause a specific effect [1–3]. Surprisingly little is known about the biophysical and biochemical interactions that take place at the cuticle to facilitate uptake of insecticides [4]. The complex and distinct physicochemical requirements for clearing each of the biological barriers that an insecticide encounters present a significant, cumulative hurdle for successful delivery of new insecticides. Compounding this problem, vertebrate animal toxicity and environmental contamination concerns may limit the applicability of an otherwise promising candidate active ingredient. Certain classes of insecticides can cause environmental contamination of soil and water resources, non-target toxicity, and have been linked to a wide array of human maladies [5,6].

Micro-and nanoparticle-based drug delivery vehicles could help overcome these limitations of existing insecticide technologies. These carriers sequester an active ingredient payload, navigate by passive or active targeting to the payload’s target site, then gradually release the payload where it is most effective. By this mechanism, such particle-associated insecticides may reduce the amount of active ingredient needed to kill pests and/or reduce the risk of environmental contamination by pesticides and associated vertebrate toxicity [7]. Biodegradable polyanhydride particles [8,9], synthesized from copolymers of 1,8-bis(*p*-carboxyphenoxy)-3,6-dioxaoctane (CPTEG), 1,6-bis(*p*-carboxyphenoxy)hexane (CPH), and sebacic acid (SA) monomers, represent promising delivery technologies for novel insecticides. These biocompatible carriers [10,11] encapsulate small molecule, nucleic acid, or protein payloads within a hydrophobic (or amphiphilic) polymer matrix, react with water to hydrolyze their anhydride bonds, and gradually release their payload as the polymer degrades [12–14] The payload release kinetics of these carriers can be tuned from hours to several months based on polymer degradation kinetics, device size, and understanding of polymer-payload interactions [15–19].

A key capacity of nano- and micro-sized carriers is their ability to overcome or circumvent biological barriers [20, 21]. It is understood that both polymer chemistry and particle size drive differential uptake kinetics, persistence kinetics and biodistribution patterns in mammals, which in turn impact payload effectiveness [22–27]. However, relatively little is known about particle uptake kinetics and biodistribution in arthropods, and only limited explorations of micro- and nanoparticle-based delivery of insecticidal agents have been performed [21,28,29]. More thorough examination of the impact of carrier polymer chemistry and size on these biological factors is necessary to maximize the potential of these technologies to deliver current and future insecticidal active ingredients.

The goal of this study was to assess transit of polyanhydride micro- and nanoparticles across the cuticle, and characterize their localization within pharmacologically important tissues in an insect pest species. We used adult female *Aedes aegypti* (Linnaeus) mosquitoes as our insect system of choice due to its public health importance as a vector of viruses that cause significant disease and mortality globally. Particle uptake and biodistribution were explored both *in vitro* in mosquito cell culture, and *in vivo* by exposing adult mosquitoes to rhodamine B (Rho)-labeled polyanhydride particles via three field-relevant routes (treated-surface contact, topical application, and exposure to treated food).

## Materials and methods

### Mosquito rearing

*Ae*. *aegypti* (Liverpool) adults were maintained in standard colony cages at 27°C (70% relative humidity). Solutions of 10% sugar water were provided to adult mosquitoes *ad libitum*. A blood source (Defibrinated sheep’s blood from Hemostat Laboratories, Dixon, CA) was supplied via an artificial membrane feeding system [30]. Four days after bloodfeeding, eggs were collected from cages and saved until hatched. Eggs were hatched in deionized water and larvae were fed Tetramin Tropical Flakes Fish Food (Tetra, Blacksburg, VA) based on larval instar and density. Male and female pupae were separated based on size. Adults were kept in 12-ounce deli cartons in densities of 50 per carton until needed. Female adults were fed 10% sugar water ad libitum until utilized for experiments. Adult mosquitoes were subjected to treatments at 3–7 days post-eclosion.

### Polymer synthesis, functionalization, and characterization

SA and Rho were purchased from Sigma Aldrich (St. Louis, MO). Triethylene glycol, 4-*p-*hydroxybenzoic acid, 1- methyl-2-pyrrolidinone, and 1,6-dibromohexane were purchased from Sigma Aldrich for CPTEG and CPH monomer synthesis. Potassium carbonate, dimethyl formamide, toluene, acetonitrile, acetic acid, sulfuric acid, N,N-dimethylacetamide, and acetic anhydride were purchased from Fisher Scientific (Fairlawn, NJ) for monomer and polymer synthesis. 4-*p*-fluorobenzonitrile was purchased from Apollo Scientific (Cheshire, UK) for use in monomer synthesis. Methylene chloride, pentane, and hexanes were purchased from Fisher Scientific for polymer purification and nanoparticle synthesis. Nuclear Magnetic Resonance (NMR) analysis used deuterated chloroform purchased from Cambridge Isotope Laboratories (Andover, MA). Rho quantification used UV-transparent microplates from Greiner Bio-One (Kremsmünster, Austria), and HPLC grade chloroform and methanol from Fisher Scientific. CPH and CPTEG diacids were synthesized as previously described [31–34]. 20:80 CPH:SA and 20:80 CPTEG:CPH copolymers were synthesized by melt condensation to number average molecular weights (M_n_’s) of approximately 17.5 and 7.5 kDa, respectively [31–33,35]. Copolymer composition and M_n_ were confirmed by ^1^H NMR analysis using a Varian MR-400 (Varian, Inc. Palo Alto, CA).

Rho was chemically conjugated to the end groups of 20:80 CPH:SA and 20:80 CPTEG:CPH copolymers by melt condensation. Rho and polymer were combined in round bottom flasks at a 10:1 Rho:end group molar ratio, acetylated with excess acetic anhydride at 150°C for 30 min, and dried by rotary evaporation. The dried mixtures were reacted for 30 min at 180°C, 0.5 Torr for 20:80 CPH:SA, and at 140°C, 0.3 Torr for 20:80 CPTEG:CPH, yielding polymers chemically conjugated to Rho terminal groups by a polyanhydride bond (Rho-20:80 CPH:SA & Rho-20:80 CPTEG:CPH, respectively). Functionalized polymers were dissolved in methylene chloride and purified by precipitation in hexanes. Nuclear magnetic resonance (NMR) spectroscopy (MR-400, Varian, Inc. Palo Alto, CA) and Fourier transform infrared (FTIR) spectroscopy (Nicolet iS5 with iD7 ATR attachment, Thermo Scientific, Waltham, MA) were used to confirm functionalization. Rho fluorophore integrity was confirmed via fluorescence spectroscopy (λ_ex_ = 540 nm) in chloroform (SpectraMax M3, Molecular Devices, San Jose, CA). Attached and unattached Rho was quantified by UV-NP-HPLC (1200 series, Agilent Technologies, Santa Clara, CA). Samples were dissolved in chloroform and separated using a Zorbax Rx-SIL 5-micron 4.6x150mm column and a gradient elution from 0.1:99.9 methanol:chloroform to 90:10 over 10 min, operating at a flowrate of 2 mL/min. Absorbance was monitored at 254 nm and 540 nm to detect polymer and Rho, respectively.

### Particle synthesis and characterization

Nanoparticles were synthesized by flash nanoprecipitation, as described previously [24,36]. Briefly, Rho-functionalized polymer was dissolved in methylene chloride (20 mg/mL), homogenized via probe sonication for 30 s at 30% amplitude, and the solution was poured into a pentane bath at a 1:250 solvent:anti-solvent ratio. Pentane was held at room temperature for Rho-20:80 CPH:SA and -10°C for Rho-20:80 CPTEG:CPH nanoparticles. Nanoparticles were collected by vacuum filtration. Microparticles were synthesized by spray drying [26]. Rho polymer was dissolved in methylene chloride (10 mg/mL) and sonicated as above. Spray drying was carried out on a BUCHI B-290 Mini Spray Dryer (New Castle, DE), with aspiration at 70%, pump at 10%, air flow at 40 (~670 L/h), and inlet temperature set at 30°C or 25°C for Rho-20:80 CPH:SA particles and Rho-20:80 CPTEG:CPH particles, respectively. Scanning electron microscopy (SEM, FEI Quanta 250, Hillsboro, OR) was used to image the nanoparticles, and size distributions were calculated using Fiji image analysis software [37] and the ParticleSizer plugin script for Fiji. Zeta potential was measured using a Zetasizer Nano (Malvern Instruments Ltd., Worcester, UK).

### Treated-surface contact exposure

Five mg of microparticles or nanoparticles were introduced into 229 μL of 556 Dow Corning Silicone Oil and 1 mL of methanol and sonicated to create a stable suspension. This suspension was immediately pipetted onto 90-mm Whatman #1 filter paper. Filter papers were dried for 24 h in a dark fume hood to remove excess solvent before exposing mosquitoes. World Health Organization conical exposure arenas were used to expose live female *Ae*. *aegypti* to the particle-treated filter papers for 48 h; mosquitoes were frozen and then removed from the exposure arena to assess the total amount of particles associated with different tissues in the live mosquitoes. The exposure tubes were frozen upside-down to prevent contamination due to mosquitoes falling onto the particle-treated filter paper. Mosquitoes were also exposed to unlabeled particles (no Rho) to assess the safety of particles to live mosquitoes.

Post-freezing, mosquitoes were collected and legs were removed; bodies and legs were separated from one another to better characterize particle localization. Legs or mosquito bodies (without legs) for each treatment were pooled (10 bodies or 60 legs per pool) and homogenized in a final volume of 1 mL phosphate buffered saline (PBS). These homogenates were incubated at room temperature for 2 h in dark to promote the release of free rhodamine into 1x PBS (as particles gradually degrade in aqueous environments). Homogenates were centrifuged at 1400 rpm to pellet cellular or tissue debris, and supernatants of each sample were used to quantify released rhodamine in the homogenate. Quantification was performed using a SPECTRA Max 384 spectrophotometer with an excitation wavelength of 545 nm and an emission wavelength of 610 nm. Supernatant (200 μL) from each homogenate was pipetted into three wells of a 96-well microplate. Fluorescence was recorded for each well and average fluorescence was used to calculate the total amount of particles localized within the legs of each mosquito (using the standard curve to calculate this amount). Ten mosquitoes were used for each replicate. This experiment was performed in triplicate with three biological cohorts.

These exposure experiments were repeated and whole mosquitoes were collected after exposure to micro- or nanoparticles for 48 h. Specimens were frozen until they were visualized. Legs were separated from mosquito bodies. Legs, whole mosquito bodies (including internal tissues), or internal tissues alone (midgut, Malpighian tubules, and ovaries) were placed onto microscope slides from each individual mosquito exposed to particles. A drop of PBS was placed on the specimens and each was covered with a coverslip. An excitation wavelength range of 510–570 nm was used to assess particle localization within mosquito tissues. Particle presence was defined as any fluorescence that was greater than the background autofluorescence defined from a control specimen (no particles). Fluorescent images and brightfield images were taken to define the boundaries of specific tissues. Microscope gain was set to 3.4x and exposure was set to one second. *Post hoc* brightness reduction was set using ImageJ analysis software (Min: 20, Max: 190). Mean fluorescence intensity (MFI) beyond background auto-fluorescence was calculated post-hoc using ImageJ. This was done by randomly highlighting labeled regions within each image and quantifying the average fluorescent signal in that region with the Analyze function in ImageJ. Fluorescent and brightfield images were presented individually and as merged images using ImageJ software. A One-way ANOVA with a Bonferroni post-hoc test (α = 0.05) was used to assess statistically significant differences between particle types.

To quantify the presence of free rhodamine, we generated a standard curve using 2 mg of micro or nanoparticles introduced into 1 mL of 1x PBS and incubated for 2 h at room temperature. Successive 10-fold serial dilutions of particles were made until the final concentration of 2 ng/mL was achieved. Legs or bodies (without the legs) of 10 mosquitoes were homogenized into each tube to account for any background fluorescence or interference caused by the presence of mosquito tissues. The fluorescence of multiple samples of 200 μL of each solution and each concentration was assessed using the excitation/emission wavelengths for the detection of Rho. The linear regions of the fluorescence/concentration curves for each particle size and chemistry were used for the standard curve regressions.

### Topical application exposure

Because microparticles did not adequately label internal tissues, only nanoparticles were used for this study. Five mg of Rho-labeled nanoparticles of either chemistry were introduced into 229 μL of 556 Dow Corning Silicone Oil and 1 mL of methanol and sonicated to create a stable suspension. This suspension was used for topical applications on mosquitoes. A slightly modified World Health Organization protocol was utilized for topical applications of nanoparticles on mosquitoes. Female, non-blood fed mosquitoes were anesthetized for 30 s using CO_2_ and then placed in a 90-mm petri dish on ice to prevent reanimation during the experiment. A volume of 0.2 μL of the nanoparticle suspension was applied to the pronotum of each individual mosquito. A total of 25 mosquitoes were treated for each experiment for each replicate. Mosquitoes were then transferred to an 8-ounce deli carton with tulle placed over the top to prevent escape and maintained at a constant temperature of 30°C, 80% relative humidity.

Whole mosquitoes were visualized using an epifluorescent microscope (excitation wavelength of 510–570 nm) to identify regions that were heavily stained with rhodamine. Finally, internalization and localization of nanoparticles in internal tissues was characterized by dissecting and visualizing internal organs of mosquitoes. The same protocol was used as described above, but with a gain setting of 1.7 X and an exposure time of 200 ms. Again, mean value fluorescence was recorded for random samples, as described in the previous section. A group was treated with soluble Rho (not linked to polymer) to control for biodistribution of soluble label. Rhodamine was estimated to be present at 13.3% and 5.2% (w/w particles) for CPTEG:CPH and CPH:SA, respectively, as the total amount of rhodamine present within each nanoparticle type. These values are calculated from the theoretical yield of rhodamine content in each nanoparticle type and represent high level estimates. The corresponding amounts of Rho for each particle type was applied to mosquitoes to assess Rho labeling in the absence of particles. A One-way ANOVA with a Bonferroni post-hoc test (α = 0.05) was used to assess statistically significant differences between particle types.

Blank nanoparticles (without rhodamine) were applied to the pronotum of mosquitoes to assess safety of nanoparticle exposure. Mosquito survival was monitored for 8 days after treatment with nanoparticle suspension.

### *Per os* exposure

Particles were incorporated into dry sucrose at a concentration of 3 mg of particles per gram of sucrose. Nanoparticle/sugar mixtures were homogenized using a mortar and pestle. After particles were evenly incorporated into dry sugar, dry sugar/nanoparticle mixtures were placed in small weigh boats and placed at the bottom of 8-ounce deli cartons. Adult, female 2–5 day old *Ae*. *aegypti* were CO_2_-anesthetized and introduced to deli cartons in groups of 10 per treatment, per replicate. Mosquitoes were provided cotton pads that were moistened with de-ionized water only. Mosquitoes were held for 48-, 120-, or 240-hr before being frozen, dissected, and assessed for Rho labeling in internal tissues. A starved control (no dry sugar) was run concurrently throughout the experimental interval to assess mortality. This was done to characterize the toxicity of particles and to better understand whether mosquitoes were feeding on sugar/particle mixtures. Mosquitoes exposed to dry sugar alone were used as controls for the dissection experiments. Examples of these mosquitoes are labeled as “controls” in figures demonstrating nanoparticle localization in internal tissues.

Images of specific tissues dissected from mosquitoes were processed using ImageJ v 2.0.0 suite (Durham, N.C.). Gain was set to 1X and exposure to 100 ms for this set of experiments to better highlight differences between treatment groups and subtract out background fluorescence. Post-hoc brightness in the red channel was set to a minimum of 20 and a maximum of 190, in order to visualize differences among treatment groups. Random samples from each treatment group were chosen and regions of stained fluorescence in the red channel was highlighted. Fluorescence intensity was measured using the Measure feature in the Analysis menu of ImageJ. The average of Mean Value Fluorescence for three samples was reported with standard error of the mean as in previous routes of exposure.

A safety study was performed to assess the toxicity of nanoparticles to exposed mosquitoes. This study was performed identically to the aforementioned study design, except that nanoparticles were not labeled with rhodamine. We also included a sugar-only control and a starved control to assess survivability of mosquitoes that did not feed.

### Cellular uptake of nanoparticles

*Ae*. *aegypti Aag2* cells were incubated at 28°C in T-25 cell culture flasks at a constant temperature of 28°C under normal atmospheric conditions. Cells were cultured in Liebovitz’s L15 media with 10% fetal bovine serum, 1% penicillin-streptomycin, and 1% L-glutamine. Cells were passaged twice per week and allowed to achieve approximately 90% confluency before each passage.

Cells were seeded on coverslips in a 24-well Corning Costar plate at a density of 5 x 10^5^ cells/well and incubated for 24 hr. Particles were introduced into each well at a concentration of 200 μg/mL and incubated for either 2 or 24 h for each nanoparticle chemistry type. After the incubation period, cells were rinsed four times with 1X PBS. After the rinse stage, cells were fixed using 4% formaldehyde solution in PBS for 10 min at room temperature. Fixed cells were washed three times with PBS and permeabilized with 1% Triton X-100 in PBS for 3 min. Cells were incubated in 2.5% AlexaFluor 488- phalloidin (Life Technologies, NY) solution in 1X PBS for 20 min. Cells were once again washed 3 times in 1x PBS and coverslips removed. These coverslips were mounted on slides using Prolong Gold reagent with DAPI (Life Technologies, NY). Mounted coverslips were coated with clear nail polish to prevent desiccation of the sample and stored at 4°C before imaging.

### Pharmacological inhibition of uptake by mosquito cells

C6/36 cells were cultured as described above. Endocytosis inhibitors were incubated with cells for 3 h prior to the addition of particles to aid in the characterization of the mechanism of cellular uptake of nanoparticles. Inhibitors were incubated with cells at similar concentrations as described in Lee *et al*. [38]. Dynasore (2.5 μM, 25 μM, and 100 μM), monodansylcadaverine (50 μM, 150 μM, and 250 μM), and nystatin (5 μM, 20 μM, and 40 μM) were used to inhibit clathrin-dependent endocytosis, receptor-mediated endocytosis, and caveolae-mediated endocytosis, respectively. Particles were then added for 2 h and cells were rinsed and fixed in the same manner as described above. A Student T-test (α = 0.05) was used to assess statistically significant differences among treatment groups.

Epifluorescence microscopy was used to produce a semi-quantitative characterization of the total percentage of cells associated with nanoparticles. This was performed by viewing 10 distinct fields-of-view (FOV) (magnification of 100X) on each cover slip. Within each viewing region, the total number of cells were enumerated and the total number of cells associated with red fluorescence (rhodamine-labeled nanoparticles) were assessed. This percentage was averaged across the 10 FOVs for each coverslip for each nanoparticle type and exposure time. A logarithmic regression was used to plot the kinetics of association of particles with Aag2 cells. Separate wells were run in parallel with nanoparticles and cells were removed and exposed to Trypan Blue to assess the number of viable cells per well. To assess cell viability, three different regions of each well were selected and the total number of blue cells (dead) were counted and compared to the total number of cells, which provided a cell viability metric for each pharmacological treatment.

To assess particle internalization within exposed cells, cells were imaged using a Leica TCS-LSI Macro confocal microscope. Excitation/emission wavelengths were optimized to view DAPI (blue), AlexaFluor 488 (green), and Rho. These excitation/emission parameters allowed for the visualization of cell nuclei, actin, and rhodamine-labeled nanoparticles, respectively. Representative images for each combination of cell type, nanoparticle type, and exposure time were obtained. Images of intracellular localization were prioritized whenever possible. Z-stacks were obtained for select representative cells and Imaris 9.1 software (Zurich, Switzerland) was used to produce 3-dimensional renderings of select cells to demonstrate particle internalization.

## Results and discussion

### Rho-labeled particle synthesis and characterization

**Fig 1:**
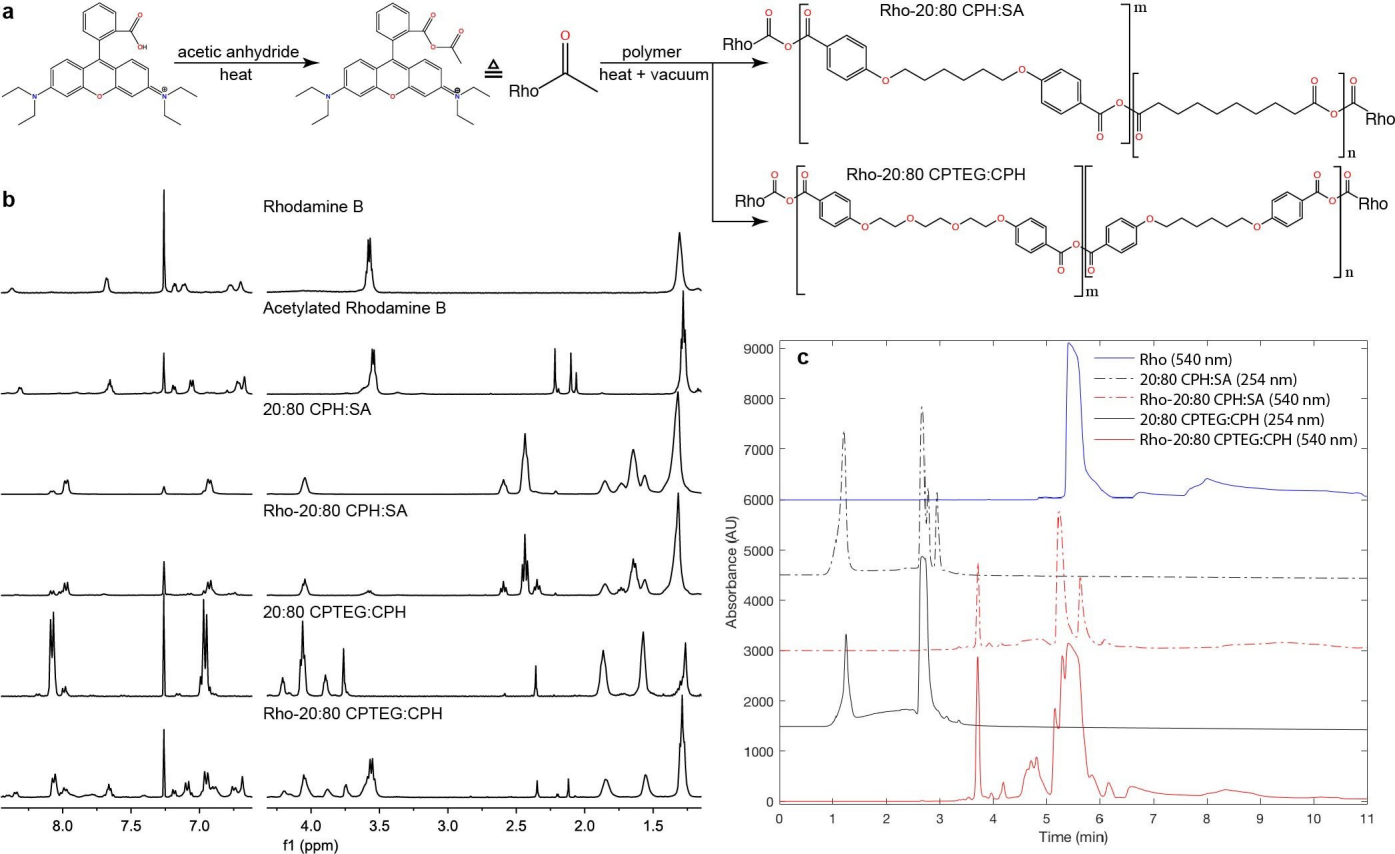
*End group functionalization of polyanhydride copolymers with Rho*. (a) Chemical synthesis scheme of Rho functionalization by melt condensation. (b) ^1^H NMR spectra of Rho-functionalized polymers and precursors. (c) NP-HPLC chromatograms of Rho-functionalized polymers for quantifying attached vs unattached Rho. Attached Rho elutes at 3.7 min and unattached Rho elutes at 5 min. Rho-functionalized polymers appear to contain significant amounts of unattached Rho.

To ensure a consistent fluorescent signal throughout the biodistribution studies, polyanhydride copolymers were end group-functionalized with a Rho fluorophore prior to nanoparticle synthesis. Functionalized 20:80 CPH:SA and 20:80 CPTEG:CPH copolymers were synthesized by a one pot, two step reaction scheme wherein the carboxylic acid group of Rho was replaced by an anhydride group, followed by covalent conjugation of Rho to the polymer with an anhydride bond via melt condensation (Fig 1A). After acetylation, Rho exhibited characteristic acetyl peaks near 2.2 ppm on ^1^H NMR spectra (Fig 1B). ^1^H-^13^C Heteronuclear Multiple Bond Correlation Spectroscopy (HMBC) NMR experiments confirm acetylation, as acetyl ^1^H’s interact with both carbonyl ^13^C’s in the anhydride group of acetylation Rho (S1A and S1B Fig). The expected HMBC peak corresponding to interaction between the nearest aromatic ^1^H with the nearby carbonyl ^13^C was not observed. This is likely due to the lower intensity of the single aromatic ^1^H compared to the three acetyl ^1^H’s, which may be lost in the noise of the experiment. The acetyl ^1^H NMR peaks disappeared almost entirely in Rho-20:80 CPH:SA and were reduced in Rho-20:80 CPTEG:CPH when compared to ^1^H NMR spectra of acetylated Rho, indicating Rho conjugation (Fig 1B). Correspondingly, the characteristic acetyl ^1^H –carbonyl ^13^C peaks disappeared in Rho-functionalized polymers in HMBC NMR experiments (S1C and S1D Fig).

Fourier-transformed infrared spectroscopy (FTIR) was used to further investigate the bonding between Rho and the polymer (S1E Fig). Rho shows characteristic peaks at 1,690 cm^-1^ and 1,583 cm^-1^ corresponding to its carbonyl and aromatic carbons, respectively. Rho-20:80 CPH:SA displays two new peaks (compared to 20:80 CPH:SA) at 1,699 cm^-1^ and 1587 cm^-1^, likely corresponding to these Rho peaks. Whereas the Rho peaks at 1690 and 1583 cm^-1^ had height ratios of approximately 2:1, Rho-20:80 CPH:SA peaks appeared at 1:1.6. This flip in peak height ratios indicates a greater prevalence of carbonyl groups relative to aromatic groups in Rho-20:80 CPH:SA compared to Rho, which is expected for a successfully functionalized polymer due to anhydride group formation (Fig 1A). Rho-20:80 CPTEG:CPH showed a new peak at 1587 cm^-1^ compared to 20:80 CPTEG:CPH, but surprisingly no peak at 1,699 cm^-1^ was observed (S1E Fig).

**Fig 2:**
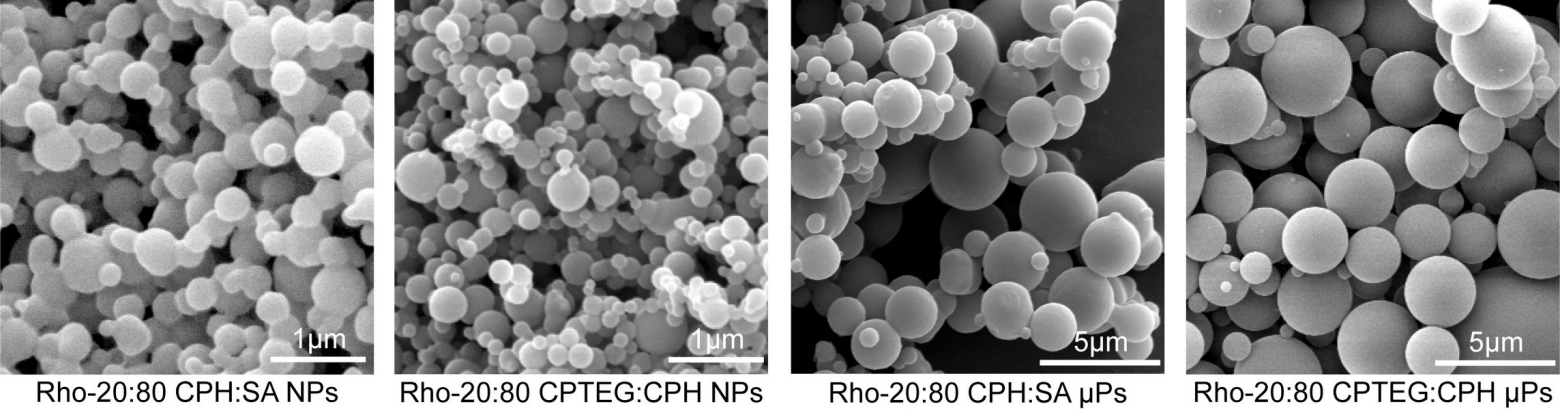
*Scanning electron micrographs of Rho-functionalized particles*. Size distributions are provided in Table 2.

Fluorescence spectroscopy experiments were performed to confirm the structural integrity of the Rho fluorophore at each step of synthesis (S1F Fig). Acetylated Rho and Rho-20:80 CPH:SA displayed similar fluorescence peak height, width, and λ_em_ maximum as the parent Rho compound, indicating that the functionalization reaction conditions did not compromise the Rho fluorophore. NP-HPLC was used to quantify attached vs unattached Rho (Table 1 and Fig 1C). In these chromatograms, unfunctionalized polymers elute between 1–3 min, functionalized polymers elute at 3.7 min, and free Rho elutes at 5 min. Notably, both Rho-functionalized polymers showed this delayed elution time compared to non-functionalized polymer, confirming functionalization. The difference in elution time between non-functionalized and Rho polymer is attributed to the higher stationary phase affinity of Rho compared to the unfunctionalized polymers. Both Rho-functionalized polymers contained approximately 2% (w/w) attached Rho (Table 1), which is comparable to the Rho (encapsulated) loading previously used in polyanhydride nanoparticles for biodistribution studies [26]. Both Rho-labeled polymers contained significant amounts of unattached Rho. Particles were also visualized via scanning electron microscopy. The sizes for all particles ranged from approximately 250 nm– 2.9 μm (Fig 2 and Table 2).

**Table 1:** Rho content of Rho-functionalized polymers quantified by NP-HPLC.

Polymer	Theoretical Attached Rho (%w/w)	Total Rho (%w/w)	Attached Rho (%w/w)
Rho-20:80 CPH:SA	5.2	14.2	2.1
Rho-20:80 CPTEG:CPH	13.3	25.6	1.9

**Table 2:** *Rho-functionalized particle characterization*.

Formulation	Diameter	Zeta Potential (mV)
Rho-20:80 CPH:SA NPs	320.8 nm ± 110.5 nm	-28.1 ± 0.361
Rho-20:80 CPTEG:CPH NPs	253.8 nm ± 96.9 nm	+33.8 ± 1.95
Rho-20:80 CPH:SA μPs	1.39 μm ± 0.83 μm	-32.1 ± 1.10
Rho-20:80 CPTEG:CPH μPs	2.87 μm ± 1.24 μm	+50.3 ± 0.802

### Treated-surface exposure

To characterize the distribution of particles in insect tissue, we first exposed mosquitoes for 48h to Rho-functionalized particles applied to a filter paper in a WHO conical exposure arena. This was done to mimic exposure to specific contact insecticides. Indoor residual spray (IRS) campaigns are an essential operational approach to control vector mosquito species in dwellings, especially in areas where malaria is endemic [39]. After an IRS application, insects that land on the treated surface become intoxicated and die. The treated-surface contact exposure route utilized in this study explores the potential of particles to associate with a surface, then translocate across the cuticle and localize within mosquito tissues and may demonstrate the potential of this technology in future IRS technologies or in trap and kill methods.

**Fig 3:**
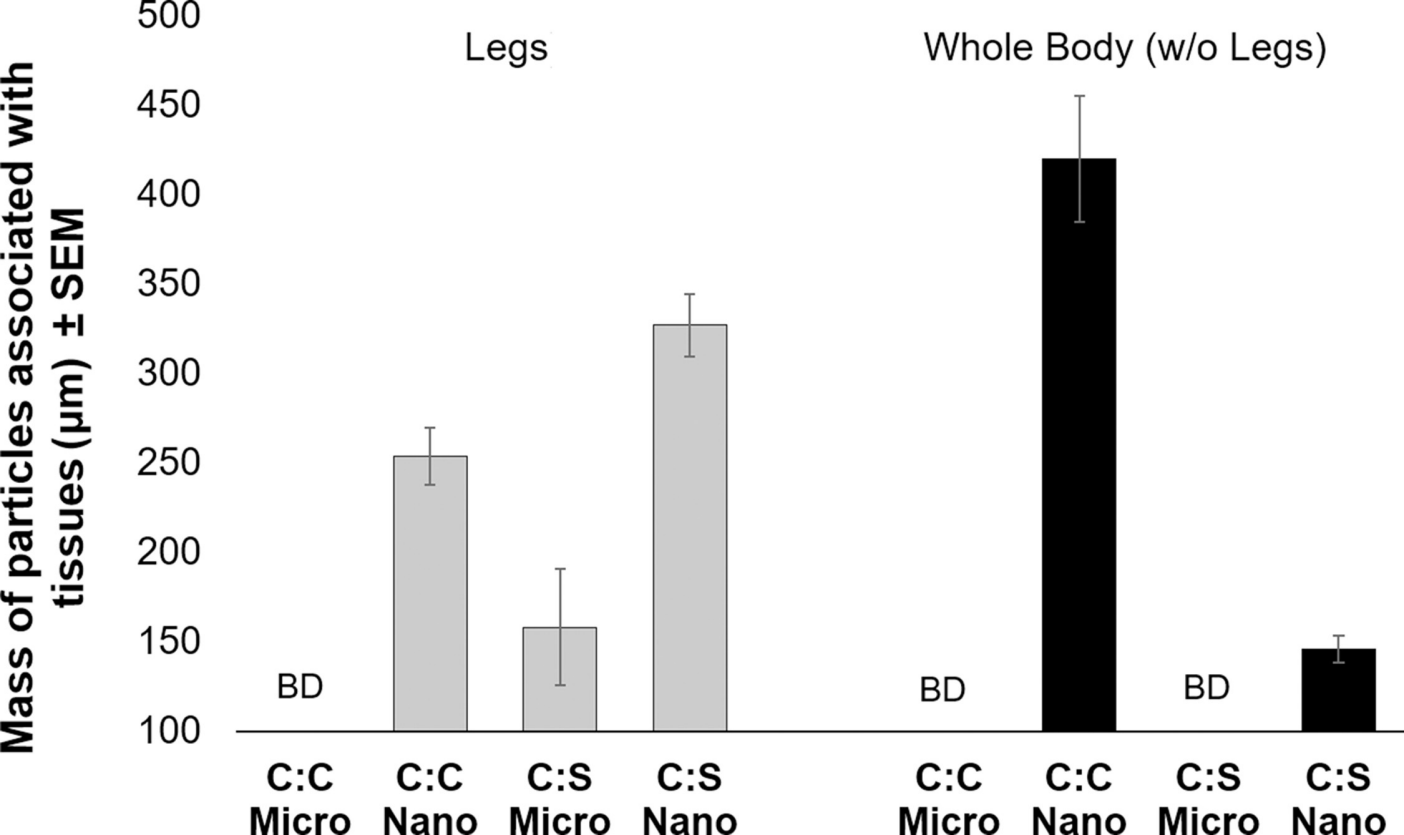
*Mass of particles associated with adult female* Aedes aegypti *legs or whole bodies (without legs) after a 48-h exposure to nanoparticles via contact with a treated surface*. Mosquitoes were collected in groups of ten and legs were removed. Legs or bodies (without legs) were then homogenized in phosphate buffered saline for 2 h, before RhodamineB content was measured for each treatment group. Standard curves were used to calculate the initial mass of particles associated with mosquitoes. CPH:SA (C:S) particles were observed in association legs than with bodies with legs removed. The opposite trend was observed for CPTEG:CPH (C:C) particles. BD = below detectability.

Using this route of exposure, particles of both chemistries were detectable at concentrations lower than 10 ng/mosquito (as measured according to a standard curve (S2 and S3 Figs)). Both microparticles and nanoparticles were associated with whole mosquito bodies (without legs) and/or legs (Fig 3). There were significant (in legs: F-statistic = 49.23, p-value = 0.0002. in whole bodies: F-statistic = 127.8, p-value < 0.0001) differences observed in localization of particles according to chemistry and particle size. The 20:80 CPH:SA nanoparticles were more often detected in leg material as compared to 20:80 CPTEG:CPH nanoparticles. It follows that 20:80 CPTEG:CPH particles more readily associated with whole body material compared to 20:80 CPH:SA. Microparticles were less likely to associate with whole bodies and legs as compared to the nanoparticles; only CPH:SA microparticles were detectable in association with legs and no microparticles were detected in whole bodies with legs removed (Fig 3). Although microparticles associated with exterior tissues to a lesser degree, larger particles might be exploited to provide a longer-lived reservoir of insecticide. Because microparticles take longer to degrade than nanoparticles, they can provide a consistent, cuticle-localized source of insecticidal payloads that can cross the cuticle without carrier help.

Because microparticles were less likely to associate with tissues in general, only nanoparticles were explored in subsequent experiments. Epifluorescence microscopy was used to track the localization of nanoparticles on the exterior cuticle of exposed mosquitoes. The 20:80 CPH:SA nanoparticles associated with all the major segments of mosquito leg, and also were observed in the posterior abdomen and the proboscis. We conclude that nanoparticles readily distribute across the exterior of the mosquito body. These findings suggest that mosquitoes may also acquire particles by contacting the surface with the tarsi or the abdomen, or by salting or probing the surface. A previous study demonstrated that mosquitoes probe and acquire solid sugar through the secretion of digestive liquids that move through the proboscis [40,41]. This same mechanism may facilitate uptake of other solids, such as the particles utilized in this study. This is a provocative lead for developing these particles for delivery of oral toxicants by surface-contact applications, perhaps in addition to topical insecticides.

**Fig 4:**
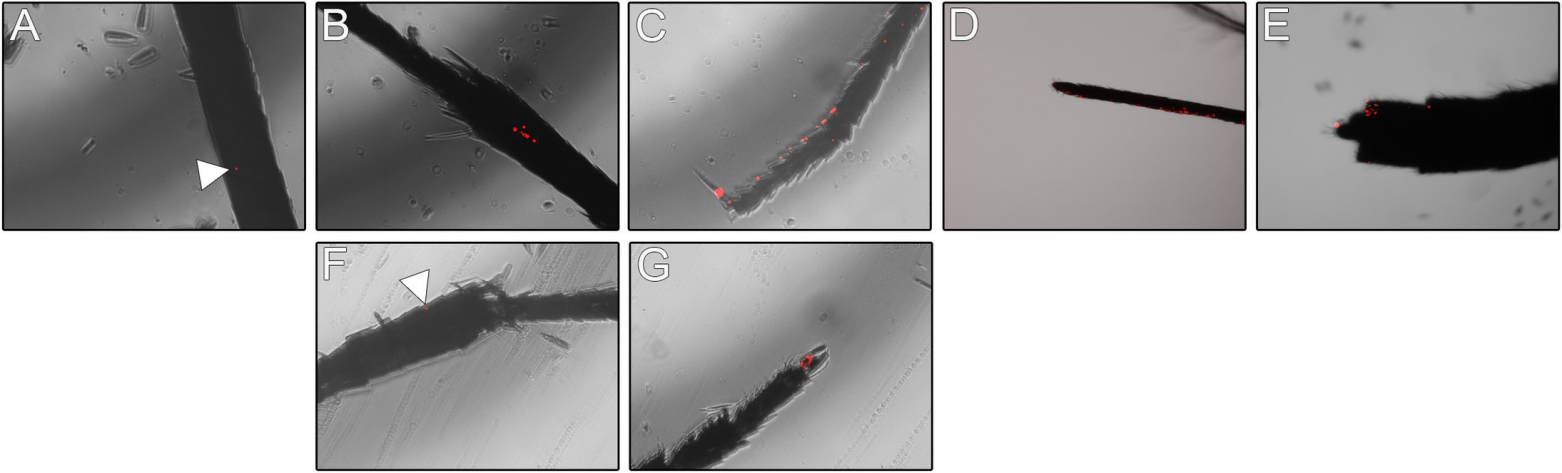
*Representative images of* Aedes aegypti *external structures associated with Rho-labeled CPH*:*SA and CPTEG*:*CPH nanoparticles following surface exposure*. Mosquitoes were exposed to particles by contact with a particle-loaded filter paper in a WHO insecticide bioassay arena. Rhodamine labeled CPH:SA nanoparticles were detected in association with the A) femur, B) tibiae, C) tarsi, D) ventral proboscis, and E) ventral abdomen of exposed mosquitoes. Rhodamine labeled CPTEG:CPH nanoparticles were detected in association with tibiae (F) and tarsi (G).

In contrast, the 20:80 CPTEG:CPH nanoparticles were only observed externally at tarsi and tibiae of exposed mosquitoes, and demonstrated less pronounced labeling (Fig 4F and 4G). CPTEG:CPH nanoparticles were not apparent elsewhere on the surface of mosquito bodies. Quantification of particles indicated that CPH:SA was more associated with the external surfaces of legs than CPTEG:CPH (Fig 4A–4E vs. 4F and 4G). The finding that these particles adhered to the cuticle, and particularly with the legs or tarsi, is promising for the delivery of insecticides with auto-dissemination properties, such as pyriproxyfen. Pyriproxyfen is carried with a mosquito when she alights on a water surfaces, thereby inoculating breeding sites with a potent inhibitor of larval development [42–44]. That said, CPTEG:CPH nanoparticles also were present at high levels in whole mosquito bodies, suggesting that these nanoparticles are internalized.

**Fig 5:**
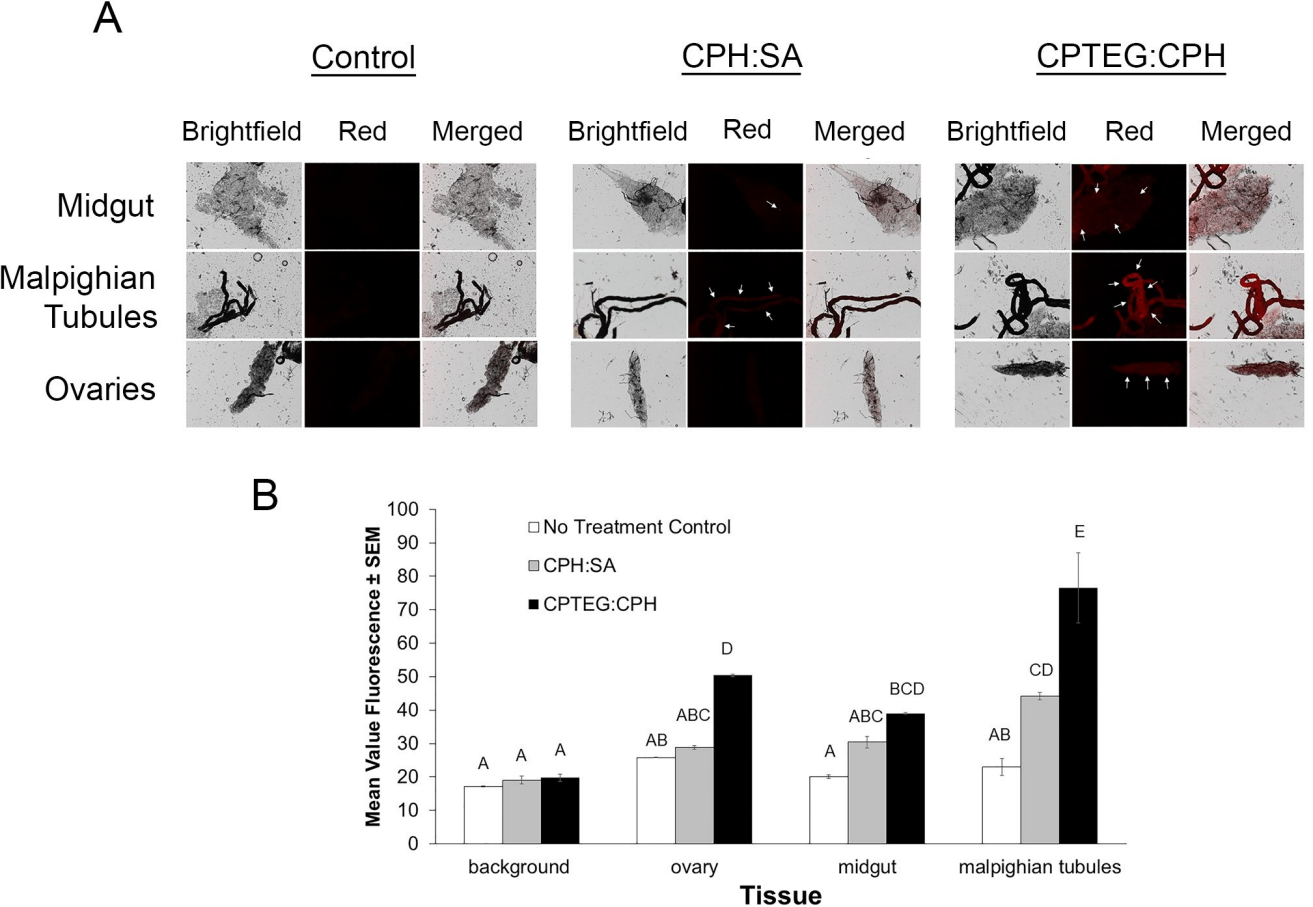
*Internal tissues labeled with both Rho-functionalized CPH:SA and CPTEG:CPH nanoparticles via treated-surface contact*. A) Rho labeling was apparent throughout various explored tissues by both nanoparticle chemistries. Arrows represent tissues with high levels of labeling compared to the control. B) Mean value fluorescence of tissue samples from mosquitoes exposed to a particle-treated surface. Letters A-D represent statistically significant differences between fluorescence intensity among various tissues and particle exposures according to an ANOVA with a Bonferroni post-hoc analysis (α = 0.05) to assess differences among treatment groups and tissues. Malpighian tubules were labeled most intensely as compared to other tissues, and CPTEG:CPH was more often observed in internal tissues, in general, compared with CPH:SA particles.

To assess internalization of nanoparticles, we dissected internal tissues and observed Rho labeling. Significant labeling of internal tissues was observed for both particle chemistries as compared to unexposed mosquitoes (Fig 5A and 5B). The 20:80 CPTEG:CPH particles were more often observed in association with internal tissues than the 20:80 CPH:SA particles, and had consistently higher mean fluorescence intensity (MFI) values (Fig 5B). Of the tissues observed, Malpighian tubules were the most significantly labeled after exposure to either particle type. These results may indicate that nanoparticles possess a higher affinity for the cells within the Malpighian tubules, or that they are actively transported there. It could also indicate that particles rapidly break down within the hemocoel of the insect and Rho dye is processed and concentrated within the Malpighian tubules. If the latter is the case, these data serve as a proxy for exploring the delivery of a small molecule payload to the Malpighian tubules, with Rho acting as an analog to an insecticidal molecule. Rho labeling was also evident within other tissues and indicates the potential for these particles to deliver toxicants to specific tissues other than the Malpighian tubules.

### Topical application

**Fig 6:**
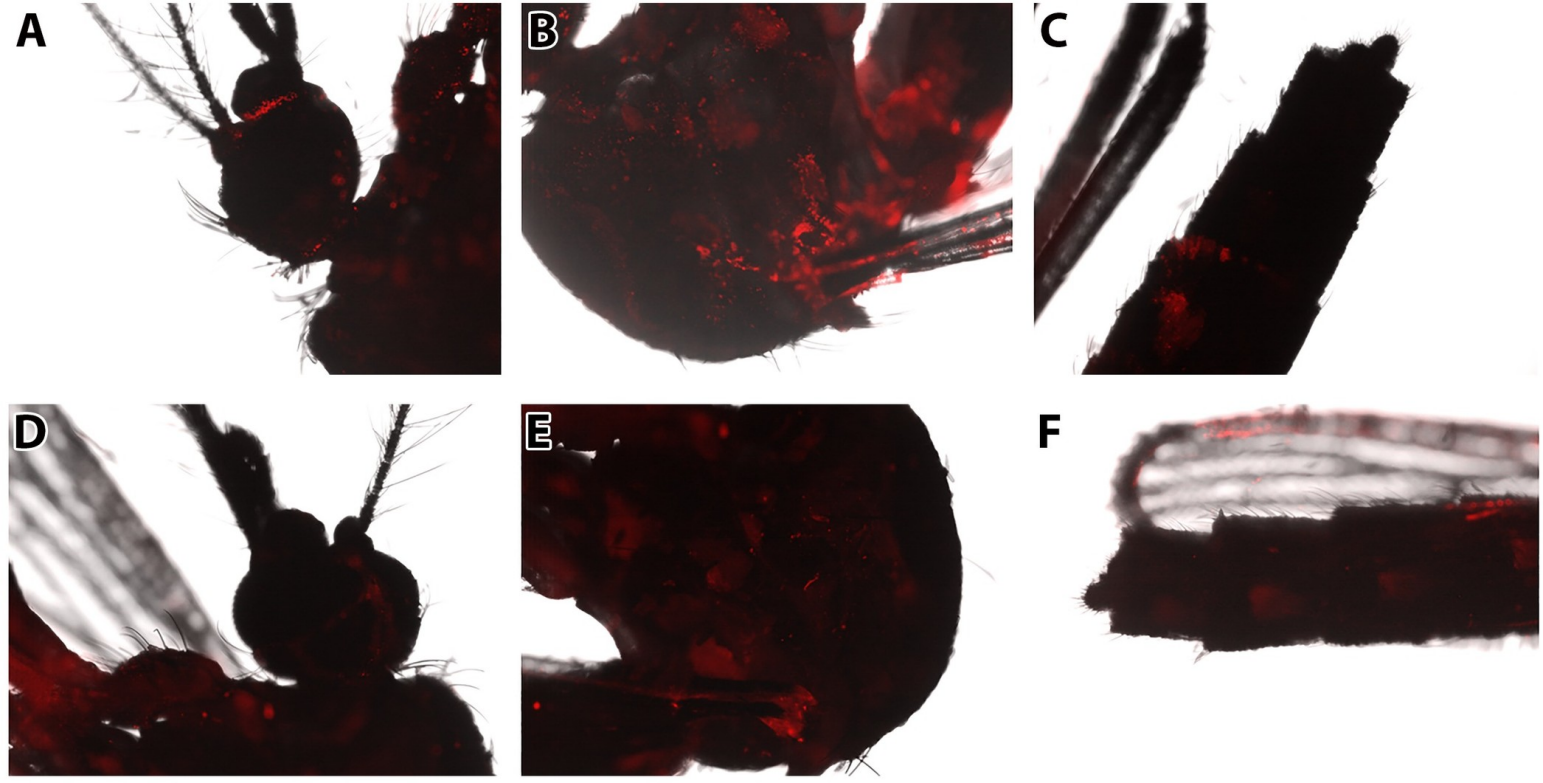
*Evidence of association of Rho-functionalized CPH*:*SA and CPTEG*:*CPH nanoparticles with the cuticle of female* Aedes aegypti *after topical exposure*. Particles were deposited in 0.2 μl volumes on the pronotum of the thorax and mosquitoes were returned to rearing conditions for 48h prior to imaging. Rho from CPH:SA nanoparticles was broadly distributed from the point of initial contact, throughout the A) head, B) thorax, and C) abdomen and was most apparent at intersegmental membranes between sclerites of the exposed insect. External labeling of mosquitoes after topical application with a suspension containing CPTEG:CPH nanoparticles was also observed in the D) head, E) thorax, and F) abdomen of mosquitoes. External Rho labeling was less intense in CPTEG:CPH trials compared with labeling observed in the CPH:SA trials. Particle labeling was diffuse throughout external tissues and even apparent on the wings of treated mosquitoes for both nanoparticle chemistries.

Topical application of 0.2 μL of particle solution to the pronotum was performed to mimic exposure to a contact insecticide application, such as space spraying or fogging a specified area. Mosquitoes then were placed in deli cartons in an environmental chamber for 48 h to allow for distribution of the nanoparticles. Because nanoparticles were the most promising at labeling internal tissues in the previous treated-surface contact studies, microparticles were excluded from topical application studies. Distinct differences were noted in the propensity of particles to label select tissues as compared to tissues from mosquitoes exposed by surface contact. Overall, both particle chemistries diffused uniformly across the cuticle surface of exposed mosquitoes post-exposure; Rho labeling was evident on the head, thorax, wings, and abdomen of treated mosquitoes (Fig 6). This distribution may be caused by grooming behavior, or by diffusion through the cuticle. The uniform distribution across exterior tissues indicates that both nanoparticle chemistries can distribute across the body of an exposed mosquito. As was observed with surface contact exposure, CPH:SA nanoparticles exhibited higher levels of Rho staining on the exterior of the cuticle than CPTEG:CPH nanoparticles. This may indicate that these particles migrate through the cuticle more slowly than CPTEG:CPH, and thus present higher levels of fluorescence when visualized on the exterior. Particle fluorescence also appeared to be greatest near the junctions of sclerites; this may indicate that nanoparticles migrate through the cuticle at these junctions, perhaps through arthrodial membranes, which are considerably thinner than other regions on the insect [45]. Further work is needed to definitively elucidate the specific route by which these particles are entering through the cuticle.

**Fig 7:**
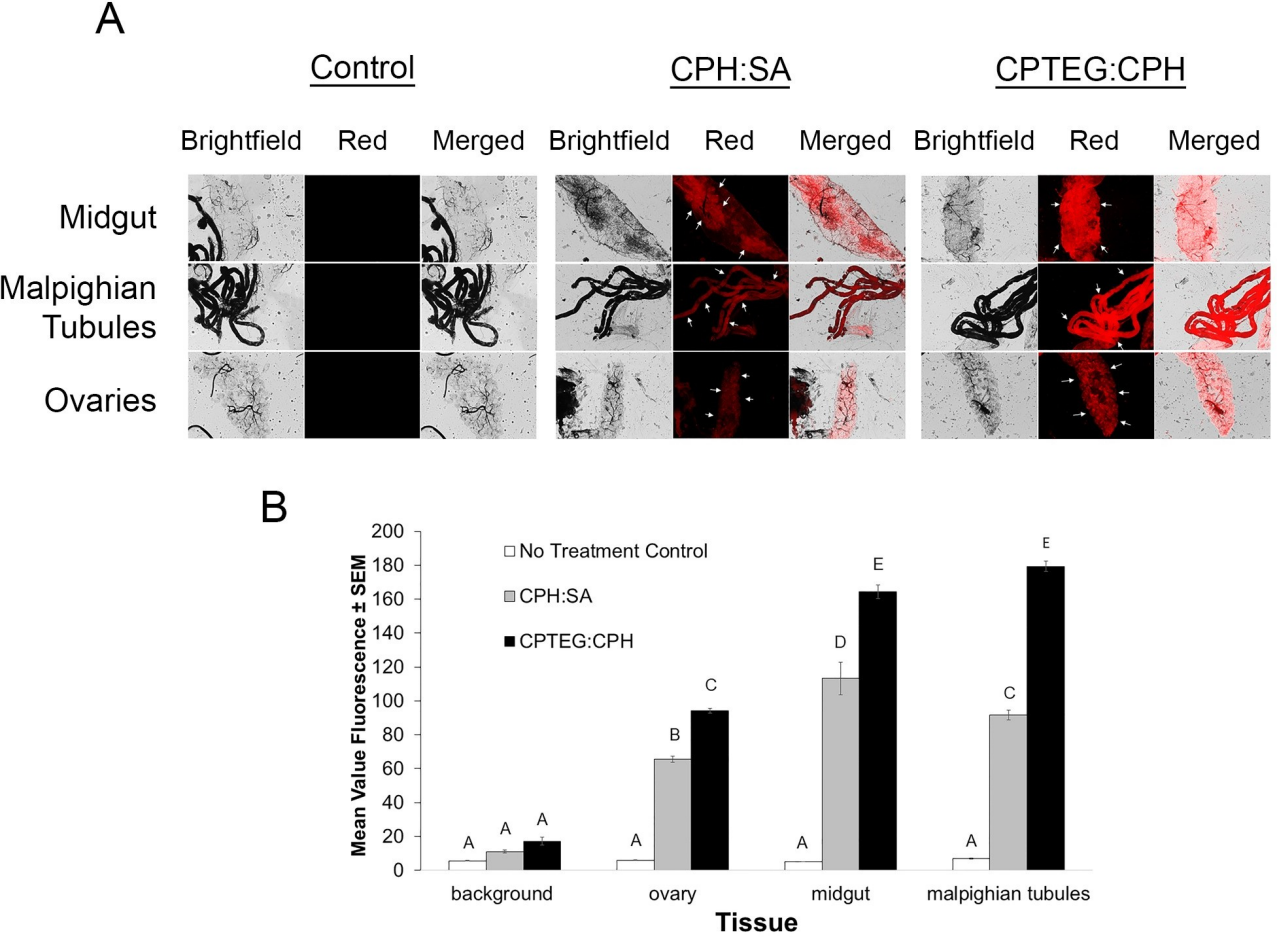
*Nanoparticle internalization of CPHSA*: *or CPTEG*:*CPH Rho labeled particle suspensions in female* Aedes aegypti *exposed via topical application*. A) Control and experimental group mosquitoes were dissected to observe midgut, Malphighian tubule and ovary tissues. Representative images are shown for each group and tissue type as they appear using bright field and fluorescence microscopy. B) Mean fluorescence intensity (MFI) was quantified for each tissue and both particle types.

Internal labeling of tissues was also observed after topical application and was significantly more pronounced than the levels obtained from the treated-surface contact exposures (Fig 7). All of the tissues characterized revealed bright Rho fluorescence compared to the controls. After 20:80 CPH:SA exposure, Malpighian tubules were the most significantly labeled, followed by the midgut and ovaries. 20:80 CPTEG:CPH particles labeled both Malpighian tubules and the midgut similarly but significantly higher than the ovaries. As with the surface contact exposure results, CPTEG:CPH Rho was more often observed in internal tissues than 20:80 CPH:SA Rho. The difference in nanoparticle uptake between exposure routes may be due to the high (1 μg of particles/mosquito) and individually-applied dose used in topical application compared to the treated-surface contact exposure. These results indicate that polyanhydride nanoparticles cross the cuticle and distribute within mosquitoes after topical application. Moreover, it is clear that the extent of biodistribution is related to the number of particles applied and the route by which mosquitoes are exposed.

The nanoparticles will release free Rho and previously attached Rho as they degrade in aqueous environments. To control for the effects of this release on tissue labeling, we exposed mosquitoes to soluble rhodamine at the levels theoretically present in each particle chemistry. No Rho association with tissues was observed in *Ae*. *aegypti* after topical exposure to Rho alone (S4 Fig). These results highlight that Rho does not migrate readily through the insect cuticle absent the aid of a nanocarrier. Therefore, Rho labeling among the particle-exposed groups is due to actual association between particles and tissues or via particle degradation and subsequent release of Rho. Both of these possibilities highlight the potential of these particles to overcome trans-cuticular transport deficiencies of an insecticidal payload.

### *Per os* Exposure

**Fig 8:**
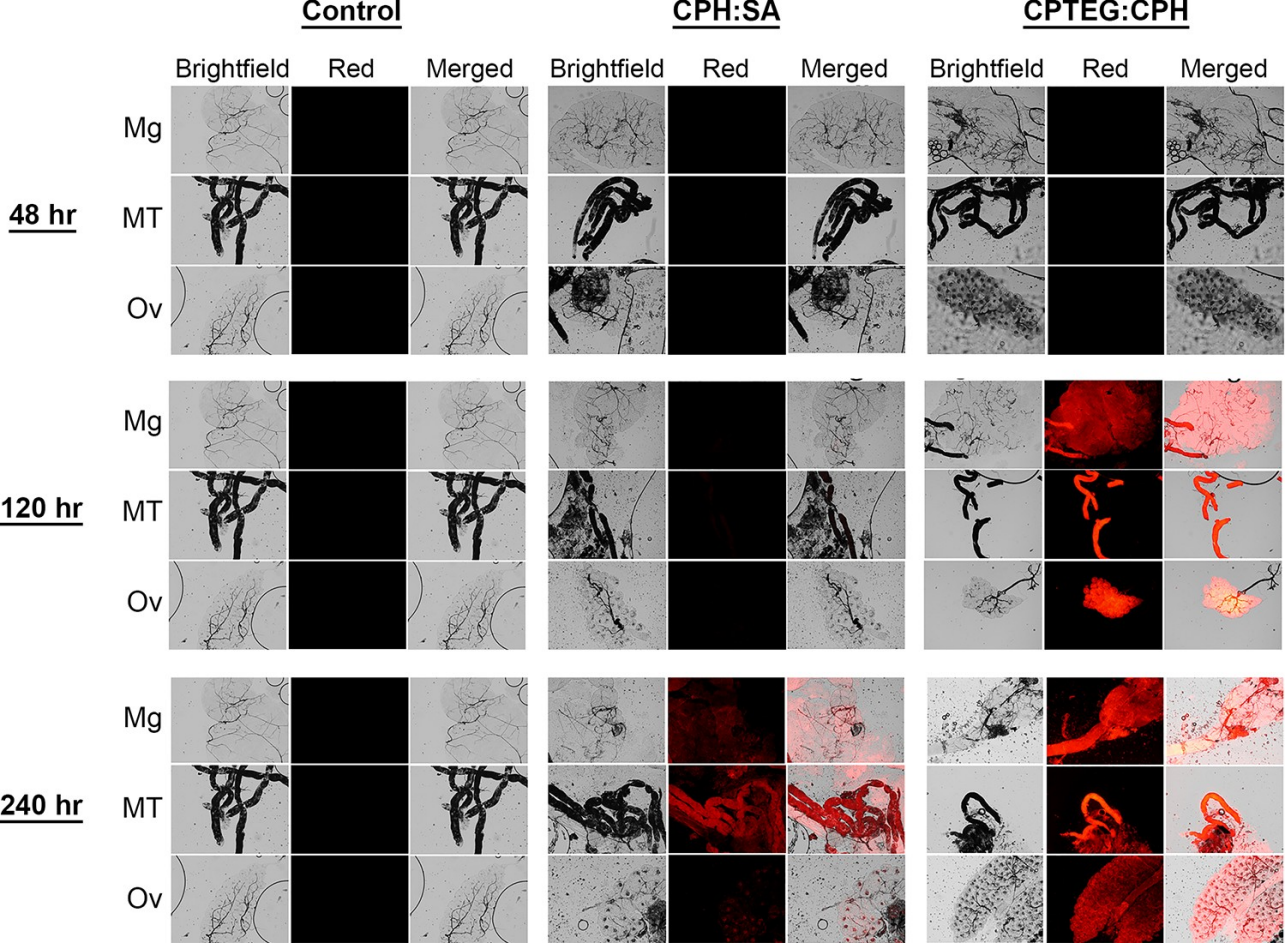
*Evidence of association of Rho-functionalized CPH*:*SA and CPTEG*:*CPH nanoparticles with the internal organs of female* Aedes aegypti *after per os exposure for 48*, *120 or 240 hours*. Control and experimental group mosquitoes were dissected to observe midgut, Malphighian tubule and ovary tissues. Representative images are shown for each group and tissue type as they appear using bright field and fluorescence microscopy.

*Ae*. *aegypti* were subjected to p*er os* exposure for 48-, 120-, or 240-h to particles in a dry sucrose:particle mixture to mimic exposure to an insecticide associated with toxic sugar baits [40,41]. Nanoparticles provided with a sugar meal disseminated and biodistributed to mosquito tissues in a chemistry- and time-dependent manner. Little-to-no Rho labeling was observed in internal tissues dissected at 48 h post-exposure (Fig 8). The low level of Rho is likely because 48 h is insufficient time for the label to reach levels that could be visualized, because *Ae*. *aegypti* tissues showed evidence of nanoparticle association and Rho labeling at 120 and 240 h post-exposure. Tissue analysis demonstrated that Rho from 20:80 CPTEG:CPH nanoparticles was more evident in internal tissues as compared to Rho from 20:80 CPH:SA. Malpighian tubules and ovaries displayed the highest Rho labeling with both particle types (Figs 8 and S10). At day 10 (240 h), Rho labeling was still apparent; however, labeling in the ovaries was considerably less that that at day 5 (120 h). As Rho labeling does not necessarily indicate nanoparticle localization, labeling may indicate that particles readily pass through the wall of the midgut or the diverticulum and distribute throughout the hemocoel, or it could indicate that particles preferentially pass into these specific tissues. These findings further underscore the ability of these particles to migrate across various biological membranes within mosquitoes and demonstrate their ability to deliver insecticidal payloads via multiple exposure routes.

Rho-only *per os* exposures were performed as controls for the treatments described above. The localization of Rho alone was apparent in various internal tissues at days 5 and 10 post-exposure (S5 Fig), but was considerably less than that present in the particle-treated groups. Rho alone was never observed in ovaries.

Overall, 20:80 CPTEG:CPH particles associated with internal and external tissues to a higher degree than 20:80 CPH:SA particles regardless of exposure route. These Rho-functionalized particles are partially positively charged based on their zeta potential. Indeed, charge is an important determinant of uptake and localization of different particle chemistries in many biological systems, tissues and cells [28]. Previous studies have demonstrated that positively charged particles were more effectively internalized in insect cells and associated with internal organs to a higher degree with select tissues than negative particles [28]. It should be noted that unlabeled polyanhydride particles typically have slightly negative zeta potentials of -20 mV [46], which may influence their effectiveness as carriers. More work is needed to characterize the contributions of particle surface chemistry and charge to the delivery of insecticides.

### Nanoparticle safety

No significant difference in survival was observed in mosquitoes exposed to particles as compared to the vehicle control or no-treatment control using any of the exposure routes tested herein. No mortality was observed until the sixth day after initial exposure to treated surfaces and mortality values were below 10% in all treatment groups (S7 Fig). Empty particles did not cause adverse effects compared to mosquitoes exposed to the carrier only until the sixth day when 20:80 CPH:SA particle-exposed mosquitoes showed a decrease in survival (80 ± 2.3%) than the control (p = 0.008) (S8 Fig). Exposure to 20:80 CPTEG:CPH particles did not result in an impact on survival via treated-surface contact, topical application, or *per os* (S7–S9 Figs). Finally, mosquitoes exposed to nanoparticles *per os* did not show any statistically significant mortality. Mortality was observed in the starved control, indicating that mosquitoes were in fact feeding on the nanoparticle-treated sugar.

### *In vitro* studies: Cellular internalization and mechanism of uptake of nanoparticles

**Fig 9:**
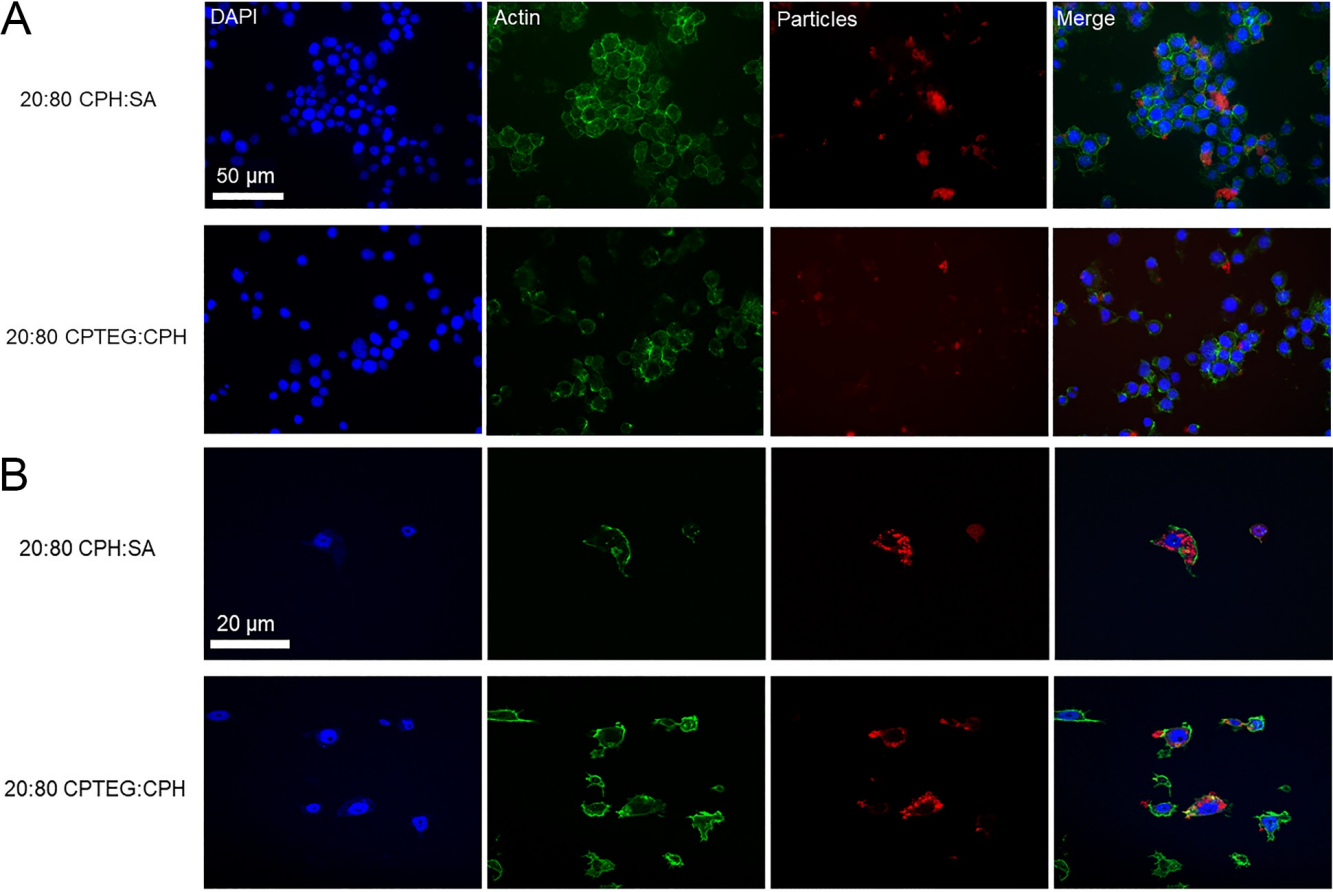
*Evidence of uptake of Rho-functionalized CPH*:*SA and CPTEG*:*CPH nanoparticles in* Aedes aegypti *Aag2 cells in culture*. DAPI was used to visualize the nucleus and phalloidin-Alexafluor 488 (green) was used to visualize the actin of the cells. Rho-labeled particles appear as red. Visualization via these methods (A: epifluorescent microscopy and B: confocal microscopy) illustrates the ability of particles to be internalized into the cytoplasm of exposed cells. Particles were not observed in association with the nuclei of exposed cells.

Internalization studies were performed to assess the ability of these particles to deliver a payload to the intracellular environment. Incubation of Aag2 cells with nanoparticles revealed rapid and robust association between particles and cells (Fig 9). After a two-hour incubation period, a majority of cells observed were positive for Rho-labeled particles (S6 Fig). The percentage of cells associated with nanoparticles increased by 20.6% (CPH:SA) and 21.8% (CPTEG:CPH) from 2 to 24 hpe. This relatively small increase over the 22 h period (2 h to 24 h exposure) indicates that a majority of nanoparticle association/intracellular localization occurs rapidly following exposure. This characteristic could be promising as these particles rapidly associate with biological tissue and deliver insecticidal molecules, even after a short exposure time.

Significant intracellular uptake was observed for both chemistry types in this study with particles inside the cells as well as adhering to the cell membrane. Both CPH:SA and CPTEG:CPH nanoparticles were efficiently internalized by Aag2 cells. No localization to the nucleus of the cells was observed. After a 24-h exposure period, Rho patterns within cells were punctate (i.e. contained in particle aggregates within cells). This staining was apparent throughout the interior of exposed cells, and a majority of cells were associated with nanoparticles (Fig 9). A 3-dimensional rendering of the internalization of nanoparticles is presented in S11 Fig. Particles can be observed throughout the interior but not in the nucleus of the cell. Overall, 20:80 CPTEG:CPH nanoparticles associated with cells to a greater degree than 20:80 CPH:SA nanoparticles (Fig 9). These characteristics of particle localization are promising and essential for the intracellular targeted delivery of small molecule payloads.

**Fig 10:**
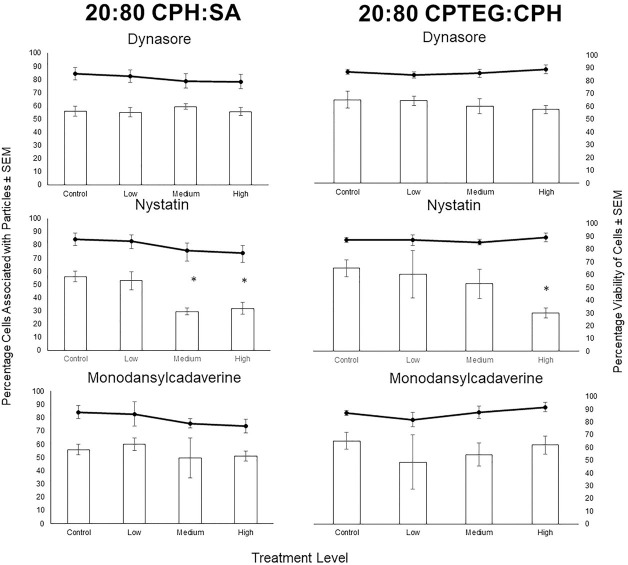
*Evidence of mechanism of endocytosis of Rho-functionalized CPH*:*SA and CPTEG*:*CPH nanoparticles in* Aedes aegypti *Aag2 using pharmacological inhibitors of clathrin-dependent endocytosis (dynasore) receptor-mediated endocytosis (monodansylcadaverine)*, *and caveolae-mediated endocytosis (nystatin)*. Cells were exposed to inhibitors for 3 h, then to particles for 2 h, and were fixed an imaged to quantify particle uptake. The percentage of Aag2 cells associated with particles ± SEM is shown with bars and the viability of Aag2 cells ± SEM is shown with lines. Dynasore and monodansylcadaverine did not produce a statistically significant effect on the association of Aag2 cells with particles. Nystatin did significantly decrease the percentage of cells that associated with both CHP:SA and CPTEG:CPH particles.

The mechanism of endocytosis of polyanhydride nanoparticles in Aag2 cells was explored using pharmacological inhibitors of clathrin-dependent endocytosis (dynasore), receptor-mediated endocytosis (monodansylcadaverine), and caveolae-mediated endocytosis (nystatin). After a 2-h exposure interval, 55.9 ± 4% of cells associated with 20:80 CPH:SA nanoparticles in the no treatment control, and 65.2 ± 7% of cells associated with the 20:80 CPTEG:CPH nanoparticles (Fig 10). This relatively high level of particle uptake by Aag2 cells in the control allowed for the characterization of uptake pathway utilizing specific pharmacological inhibitors. None of the inhibitors caused a significant decrease in cell viability in any of the treatment groups compared to the control, allowing for further identification of potential endocytosis mechanisms. Dynasore did not produce significant inhibition of endocytosis of nanoparticles, with 55.1 ± 4%, 59.4 ± 2%, and 55.5 ± 3% of cells associating with the 20:80 CPHS:SA particles for the low (2.5 μM), medium (25 μM), and high (100 μM) dose exposure to this compound. This was similar to the 20:80 CPTEG:CPH chemistry with 64.3 ± 4%, 60.2 ± 6%, and 57.5 ± 3% of cells associated with nanoparticles for the low, medium, and high exposure groups, respectively. Monodansylcadaverine (MDC) also did not produce significant inhibition of nanoparticle uptake for either chemistry at all the concentrations screened. The percentages of cells associated with the 20:80 CPH:SA nanoparticles were 60.0 ± 4.8%, 49.7 ± 15%, and 51.1 ± 4% for the low (50 μM), medium (150 μM), and high (250 μM) exposures, respectively; and for the 20:80 CPTEG:CPH nanoparticles, the respective numbers were 48.5 ± 21%, 54.5 ±9%, and 62 ± 7%.

In contrast, incubation with nystatin resulted in significant decrease in Aag2 cellular association with both types of nanoparticles. The uptake of 20:80 CPH:SA particles was inhibited significantly at the medium (20 μM) and high (40 μM) exposure levels, with 29.4 ± 4% and 31.8 ± 5% of cells associating with nanoparticles in each of these exposure groups, respectively. Nystatin also significantly inhibited the uptake of the 20:80 CPTEG:CPH nanoparticles, with a high exposure of nystatin causing only 30.0 ± 4% of cells to associate with nanoparticles compared to the 65.2 ± 7% of cells in the control. The effect of nystatin was greater on nanoparticle uptake in the context of 20:80 CPH:SA as compared to 20:80 CPTEG:CPH, as the medium dose significantly decreased nanoparticle association in the cells exposed to 20:80 CPH:SA but not 20:80 CPTEG:CPH particles. Nystatin is a known inhibitor of caveolae-mediated endocytosis [39]. This result may indicate that caveolae-mediated uptake is essential for nanoparticle internalization. Caveolae-mediated uptake is commonly associated with the uptake of small particles (i.e. <100 nm), so it is curious that this process was so important for particle uptake in this study (with a majority of our particles ranging from 100–700 nm in diameter) [47]. However, it is also possible for caveolae to take up materials that are larger than the diameter of caveolae. As caveolae are important in the transcytosis of materials across epithelial membranes [48–50], caveolae-mediated transcytosis may, in part, explain the migration of particles through epithelial barriers and into internal tissues, as demonstrated in the in vivo exposure work discussed above. Moreover, the results of this experiment demonstrate that the nanoparticles mentioned in this study may move through the basal lamina, either by passive or active transport. As a majority of these nanoparticles are above 90 nm, significantly larger than all arboviruses (50–80 nm) [51,52], these results may represent provocative findings for future studies elucidating the mechanisms of basal lamina transversal of virions and other biological or physical particles. Other endocytic pathways may also be involved in intracellular uptake as the pharmacological panel of inhibitors was not exhaustive.

## Conclusions

Nano- and microparticle carriers improve payload activity by navigating challenging biological barriers and delivering their cargo to putative sites of action [15,53–57]. We hypothesized that polyanhydride particles could function in the same way in arthropod systems to deliver insecticidal compounds. Polyanhydrides have demonstrated high biocompatibility and safety in mammalian models and humans, and are therefore attractive for use in insecticidal formulations. Given the importance of the cuticle’s role in determining insecticidal potency, it was of particular interest to test whether these particles could traverse the cuticle and localize in internal tissues of mosquitoes. This could provide a breakthrough delivery platform for a larger and more diverse suite of active ingredients that can be employed to manage populations of vector arthropod species.

Toward this end, we tested polyanhydride particle association and uptake *in vitro* and *in vivo* in the mosquito, *Ae*. *aegypti*. Firstly, a novel end group functionalization scheme was developed to persistently label particles with a fluorophore. We synthesized particles of two different copolymer compositions at two different sizes to glean information about the influence of particle physicochemical properties and size on insect cuticle migration. Both polyanhydride micro- and nano-formulations associated with *Ae*. *aegypti* cuticle after surface contact, but nanoparticles do so to a greater extent as measured by dispersal and intensity of the Rho label. Therefore, only nanoparticles were assessed for uptake and dissemination after topical and *per os* exposure. Nano-formulations based on both 20:80 CPTEG:CPH and 20:80 CPH:SA chemistries appear to traverse the cuticle and disseminate to tissues including the Malpighian tubules, midgut and ovary, but 20:80 CPTEG:CPH particles were more often observed in association with internal tissues, and with higher intensity, than the 20:80 CPH:SA particles (Fig 5). Both 20:80 CPTEG:CPH and 20:80 CPH:SA nanoparticles distributed across the cuticle of the head, thorax and abdomen of mosquitoes after topical exposure to the thorax (Fig 6), and particles disseminated to the Malpighian tubules, midgut and ovary. As was the case with surface contact exposure, 20:80 CPTEG:CPH particles were more often observed in association with internal tissues, with higher intensity. The same was true of uptake and dissemination of these nanoparticles in mosquitoes exposed to particles provided *per os* in a dried sugar meal (Fig 8). Safety studies of particle exposure revealed no untoward impact on mosquito survival as a function of exposure to the particles, nor the particles labeled with Rho. Finally, particles were readily and rapidly (within 2 h) taken up by *Ae*. *aegypti* cells in culture, so demonstrating the capacity of these particles to migrate not only into tissues, but inside the cells (Fig 10). Cellular uptake appears to be at least in part associated with caveolae-mediated endocytosis (Fig 10).

Altogether, these observations point to the capacity of these particles to navigate vector biological barriers, and prompt further exploration of the particles for use as a delivery system for active ingredients with insecticidal activity. Based on the observations herein, particle chemistry and exposure route can be exploited to target insecticidal molecules to specific sites within insects, or enable the phoretic transport of insecticide-loaded particles to other tissues. It may be possible to exploit these technologies to deliver synthetic small molecule and next-generation insecticidal active ingredients. For example, several authors have suggested coupling RNAi technologies to micro- and nano-particle delivery technologies to allow for more effective delivery to target tissues [58–60]. Moreover, nanocarriers for insecticidal small molecules could be tailored to protect cargo from environmental degradation, and/or slowly release cargo to improve persistence within the target pest.

## Supporting information

S1 Fig *Rho acetylation and Rho labeled polymer structural characterization*. (a-b) ^1^H-^13^C 2D NMR spectra of acetylated Rho using HMBC (a) and HSQC (b). Inset arrows indicate successful acetylation of Rho. (c-d)) ^1^H-^13^C 2D HMBC NMR spectra of Rho-20:80 CPH:SA (c) and Rho polymers-20:80 CPTEG:CPH (d). Acetyl peaks from the acetylated Rho (a) sample are not present in Rho labaled polymers, indicating consumption of the acetylated Rho precursor. All 2D NMR spectra have been denoised. (e) FTIR spectra of Rho labaled polymers and precursors. Inset arrows identify characteristic Rho peaks at 1,690 cm^-1^ and 1583 cm^-1^. Data have been normalized to similar peak heights and offset for visual clarity. (f) Fluorescence spectra of Rho-20:80 CPH:SA and precursors. Acetylation and end group functionalization do not appear to compromise the integrity of the Rho fluorophore.
(TIF)

S2 Fig *Standard curves of nanoparticles and microparticles in PBS with homogenized mosquito legs*. Relative fluorescence units (RFU) is plotted with respect to μg/mL of nanoparticles within solution. A and B correspond to CPTEG:CPH microparticles and nanoparticles, respectively, and C and D represent standard curves for CPH:SA microparticles and nanopartilces.
(TIF)

S3 Fig *Standard curves of nanoparticles in PBS with homogenized whole mosquito bodies (no legs)*. Relative fluorescence units (RFU) is plotted with respect to μg/mL of nanoparticles within solution. A) CPH:SA nanoparticles; B) CPTEG:CPH nanoparticles.
(TIF)

S4 Fig *Internal tissues of* Aedes aegypti *mosquitoes treated topically with soluble Rhodamine B*. Mosquitoes were exposed to Rhodamine B alone, at the concentration associated with CPH:SA and CPTEG:CPH nanoparticles (see Figs 4–8)**. **
(TIF)

S5 Fig *Internal tissues of* Aedes aegypti *mosquitoes exposed to soluble Rho* per os. Mosquitoes were exposed to Rhodamine B alone, at the concentration associated with CPH:SA and CPTEG:CPH nanoparticles (see Figs 4–8).
(TIF)

S6 Fig *Association kinetics of CPH:SA and CPTEG:CPH particles applied to Aag2 cells*. A majority of cells associate with both particle chemistries within two hours and this association increases steadily at 24 hours after treatment. The number of cells labeled with particles were enumerated on slides of fixed cells that were exposed to both particle chemistries. CPTEG:CPH particles associated with more cells than did CPH:SA particles.
(TIF)

S7 Fig *Survival of* Aedes aegypti *adult female mosquitoes exposed to blank nanoparticles (not labeled with Rho) via treated-surface contact*. Little-to-no mortality was observed throughout the 8-day exposure interval, indicating these particles are not toxic at the concentration applied in this assay. This experiment was replicated in triplicate (N = 30). A student t-test was used to compared each treatment back to the no treatment control.
(TIF)

S8 Fig *Survival of* Aedes aeygpti *mosquitoes exposed to no treatment*, *blank CPH*:*SA particles*, *or blank CPTEG*:*CPH particles via topical application*. No differences in percentage mortality were noted between the particle and control treatments until day 6. CPH:SA particles produced lower survival compared to CPTEG:CPH particles; however, this difference was not statistically significant. This experiment was run in triplicate (N = 30).
(TIF)

S9 Fig *Survival of* Aedes aeygpti *mosquitoes exposed to no treatment*, *CPH*:*SA particles*, *or CPTEG*:*CPH particles* per os. No differences in percentage mortalities were noted between the particle and Sucrose Control treatment throughout the entire experiment. The starved control produced statistically significant mortality compared to all other groups at day 5 and beyond. This indicates that mosquitoes feed on nanoparticle-sugar mixtures and they do not produce toxic effects at the concentrations utilized in this study.
(TIF)

S10 Fig *Mean fluorescence intensity of tissue samples from* Aedes aeygpti *at 48 h post exposure* per os. (TIF)

S11 Fig *Three-dimensional rendering of Aag2 cells exposed to CPTEG:CPH nanoparticles* after 24 h. Aag2 cells were exposed to nanoparticles for 24 h and rinsed with DPBS three times. Cells were then fixed to the slides and visualized with confocal microscopy. Z-stack images were combined using Imaris software and three-dimensional video was produced. Nanoparticles are localized within the cytoplasm of exposed cells and also associate with the exterior of the treated cells.
(AVI)
