# Supplementary figures and images for: Biodistribution of degradable polyanhydride particles in Aedes aegypti tissues
Source: PLoS Negl Trop Dis. 2020 Sep 8;14(9):e0008365. doi: 10.1371/journal.pntd.0008365 (PMC7500644; doi:10.1371/journal.pntd.0008365)

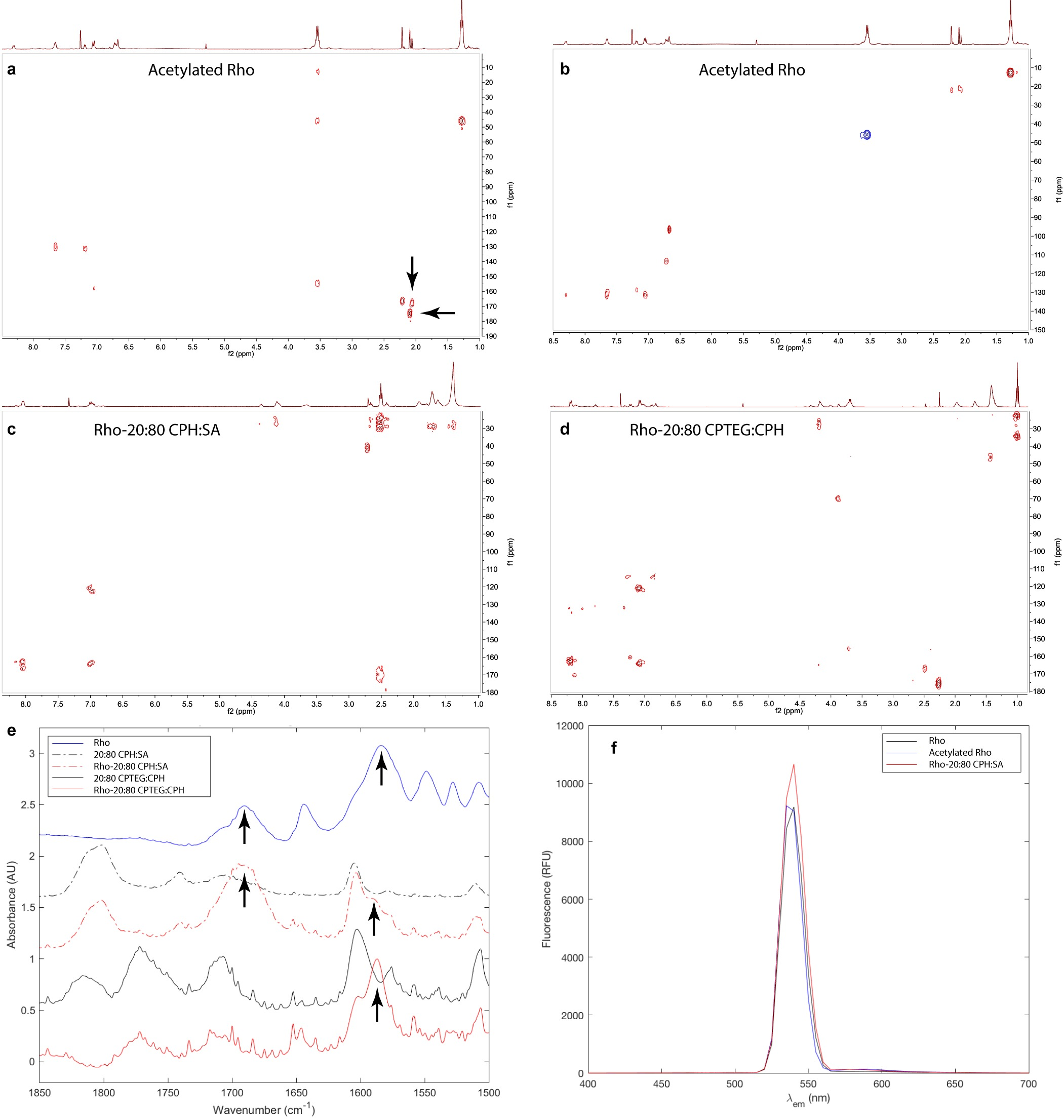

Supplement: S1 Fig — (a-b) 1H-13C 2D NMR spectra of acetylated Rho using HMBC (a) and HSQC (b). Inset arrows indicate successful acetylation of Rho. (c-d)) 1H-13C 2D HMBC NMR spectra of Rho-20:80 CPH:SA (c) and Rho polymers-20:80 CPTEG:CPH (d). Acetyl peaks from the acetylated Rho (a) sample are not present in Rho labaled polymers, indicating consumption of the acetylated Rho precursor. All 2D NMR spectra have been denoised. (e) FTIR spectra of Rho labaled polymers and precursors. Inset arrows identify characteristic Rho peaks at 1,690 cm-1 and 1583 cm-1. Data have been normalized to similar peak heights and offset for visual clarity. (f) Fluorescence spectra of Rho-20:80 CPH:SA and precursors. Acetylation and end group functionalization do not appear to compromise the integrity of the Rho fluorophore. (TIF) [file pntd.0008365.s001.tif]

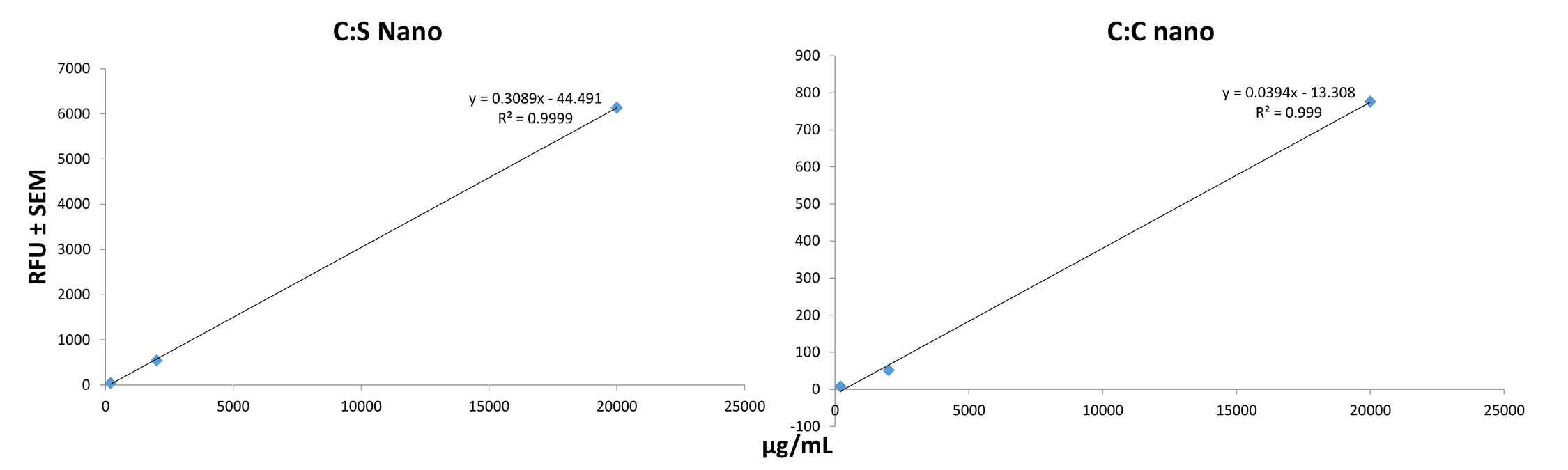

Supplement: S2 Fig — Relative fluorescence units (RFU) is plotted with respect to μg/mL of nanoparticles within solution. A and B correspond to CPTEG:CPH microparticles and nanoparticles, respectively, and C and D represent standard curves for CPH:SA microparticles and nanopartilces. (TIF) [file pntd.0008365.s002.tif]

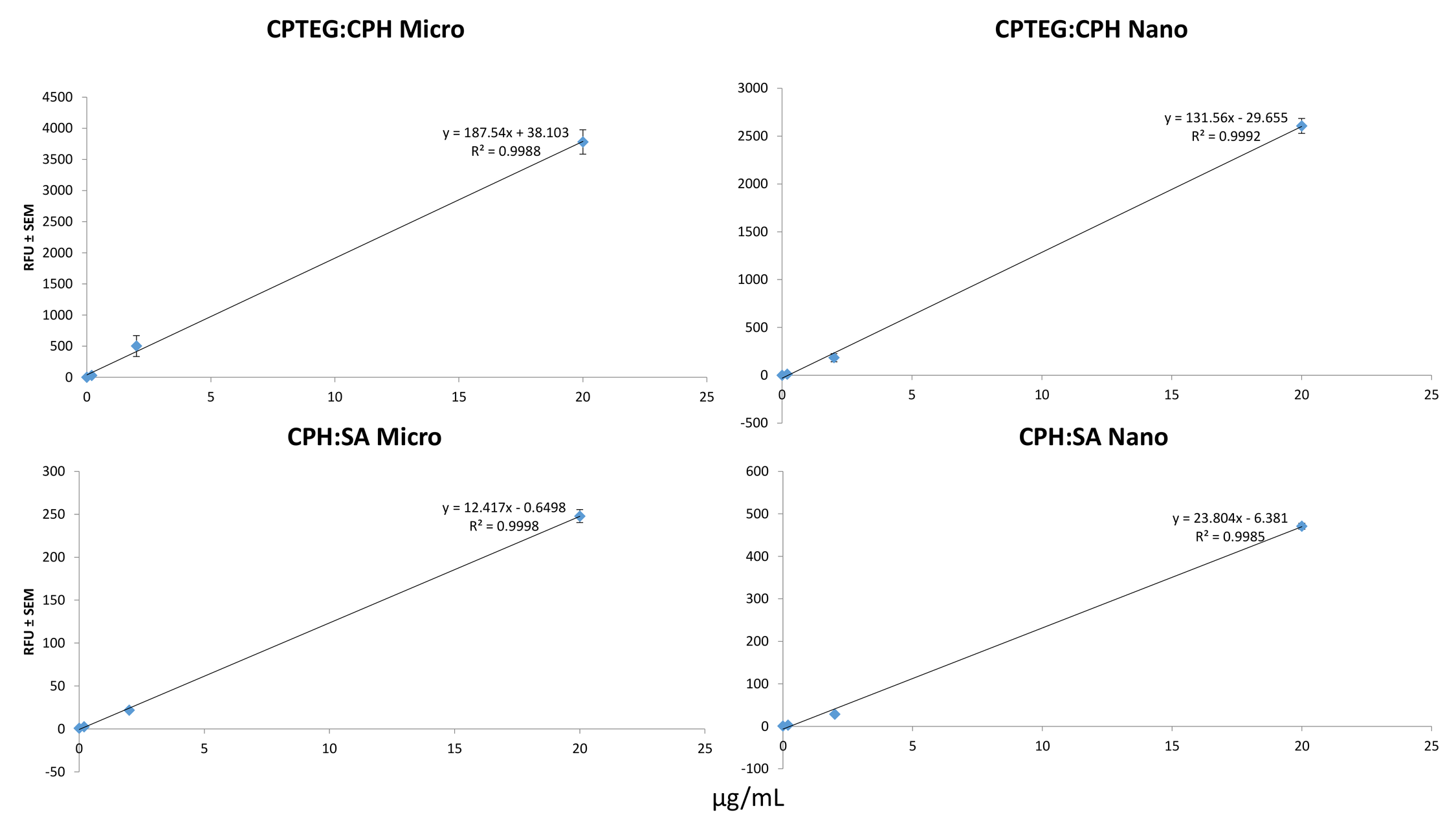

Supplement: S3 Fig — Relative fluorescence units (RFU) is plotted with respect to μg/mL of nanoparticles within solution. A) CPH:SA nanoparticles; B) CPTEG:CPH nanoparticles. (TIF) [file pntd.0008365.s003.tif]

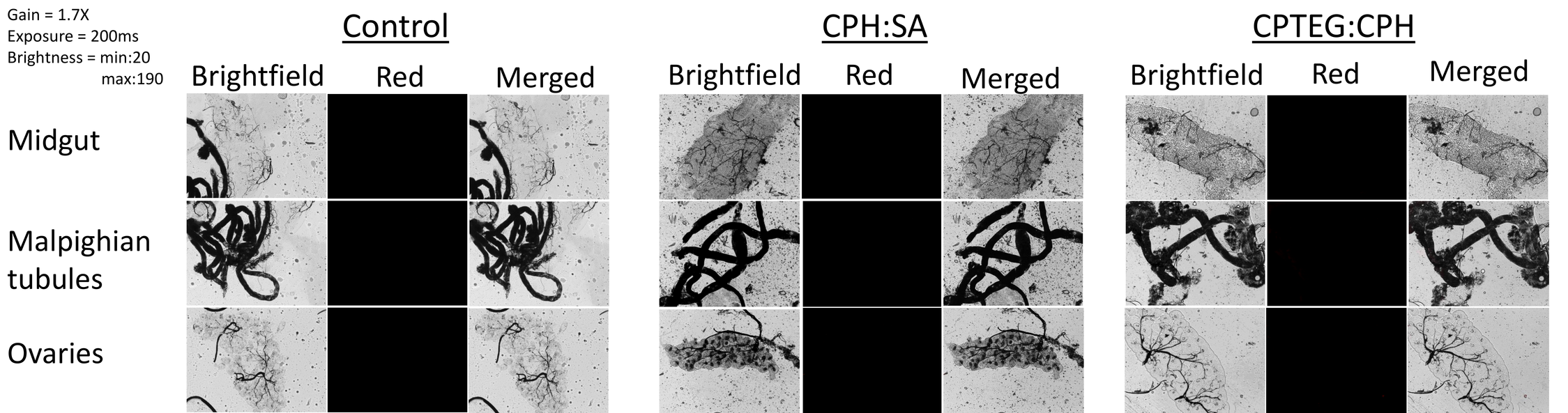

Supplement: S4 Fig — Mosquitoes were exposed to Rhodamine B alone, at the concentration associated with CPH:SA and CPTEG:CPH nanoparticles (see Figs 4–8). (TIF) [file pntd.0008365.s004.tif]

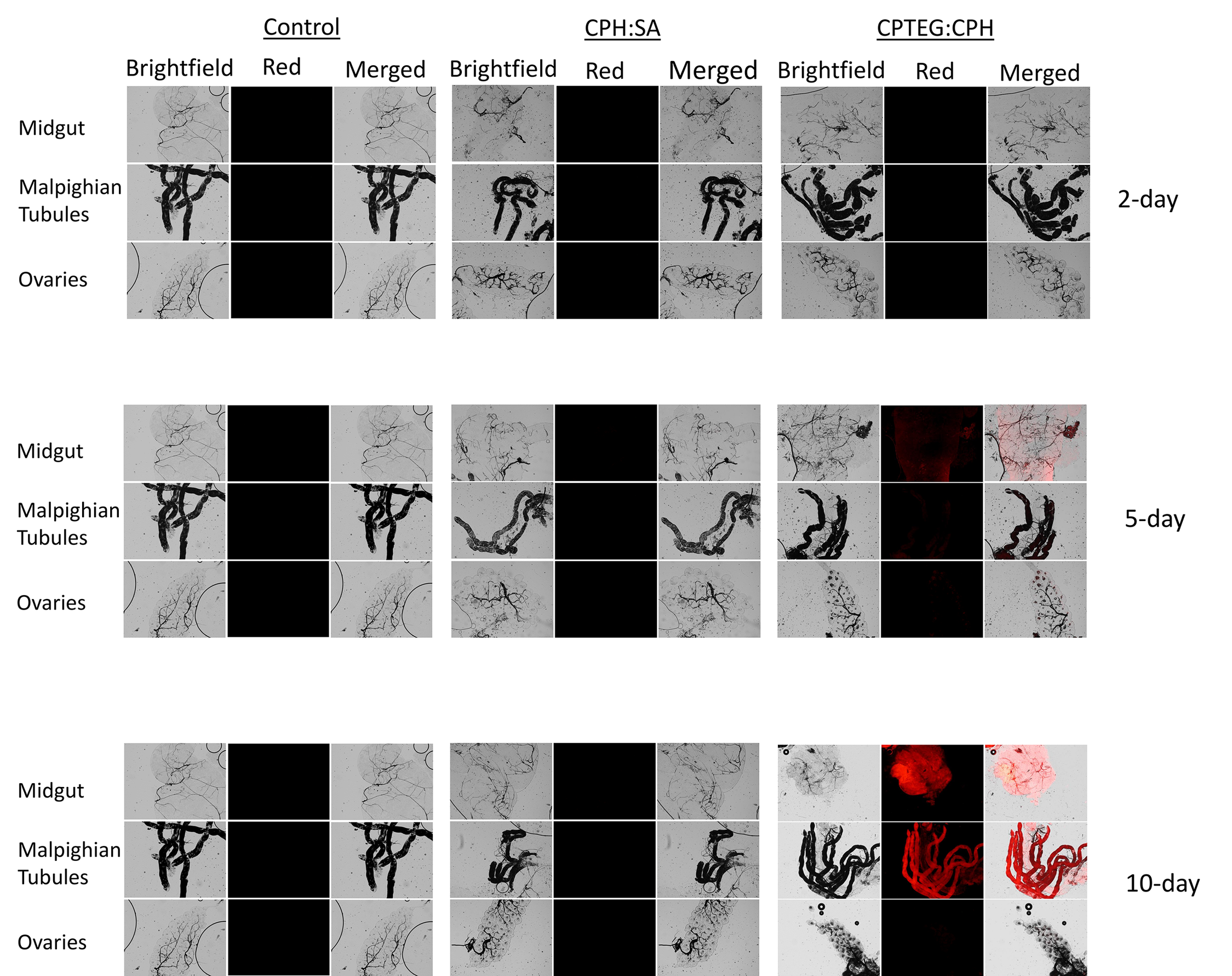

Supplement: S5 Fig — Mosquitoes were exposed to Rhodamine B alone, at the concentration associated with CPH:SA and CPTEG:CPH nanoparticles (see Figs 4–8). (TIF) [file pntd.0008365.s005.tif]

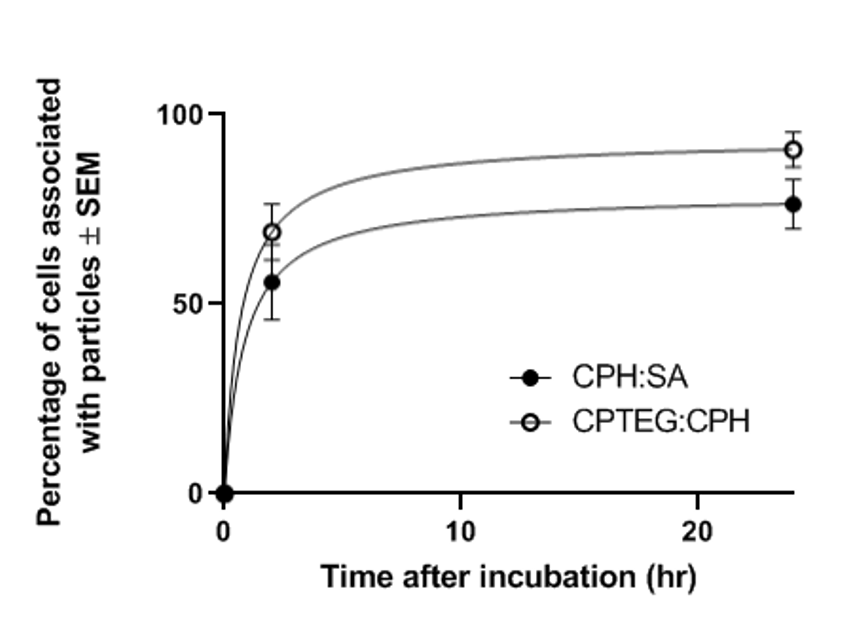

Supplement: S6 Fig — A majority of cells associate with both particle chemistries within two hours and this association increases steadily at 24 hours after treatment. The number of cells labeled with particles were enumerated on slides of fixed cells that were exposed to both particle chemistries. CPTEG:CPH particles associated with more cells than did CPH:SA particles. (TIF) [file pntd.0008365.s006.tif]

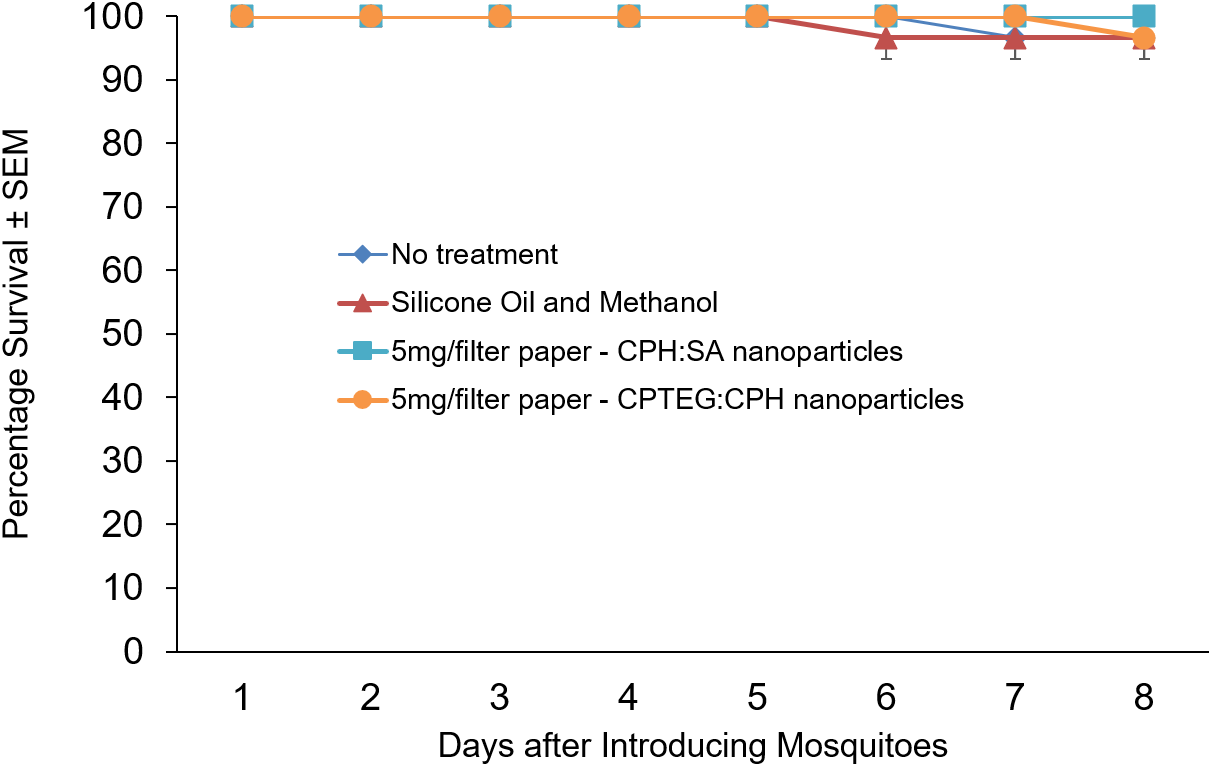

Supplement: S7 Fig — Little-to-no mortality was observed throughout the 8-day exposure interval, indicating these particles are not toxic at the concentration applied in this assay. This experiment was replicated in triplicate (N = 30). A student t-test was used to compared each treatment back to the no treatment control. (TIF) [file pntd.0008365.s007.tif]

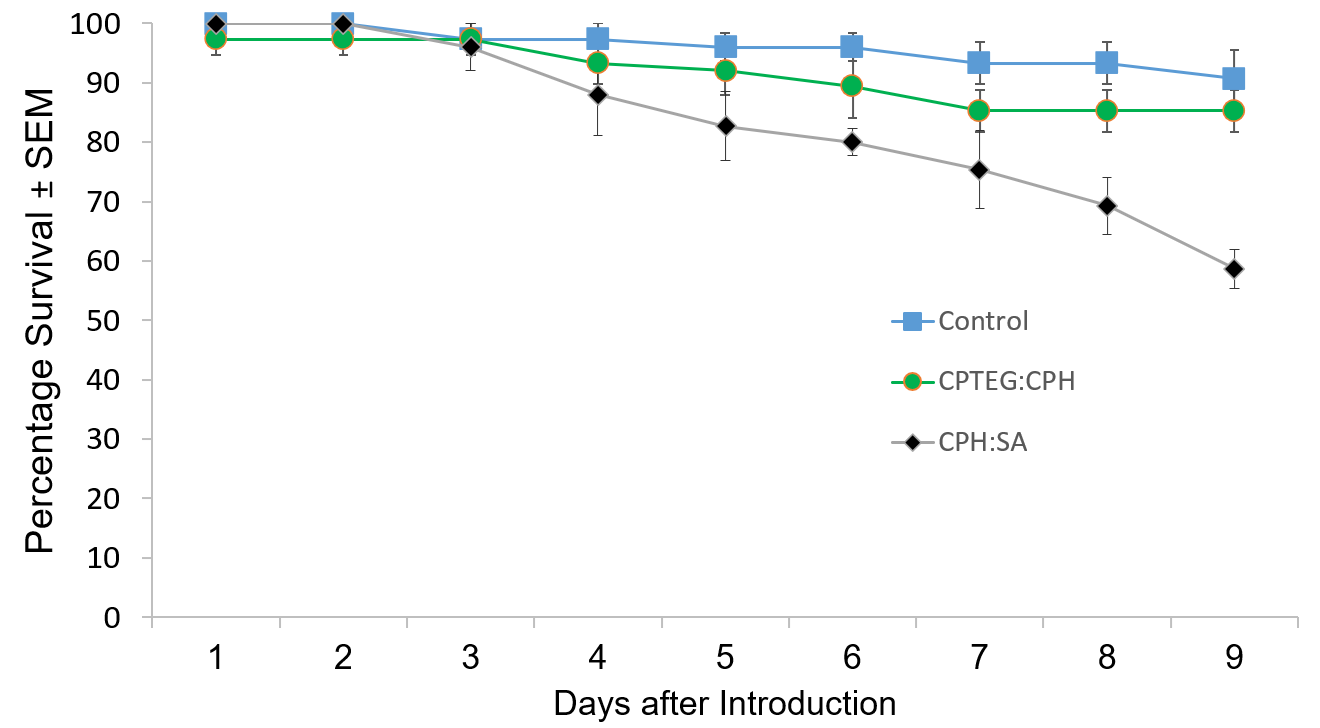

Supplement: S8 Fig — No differences in percentage mortality were noted between the particle and control treatments until day 6. CPH:SA particles produced lower survival compared to CPTEG:CPH particles; however, this difference was not statistically significant. This experiment was run in triplicate (N = 30). (TIF) [file pntd.0008365.s008.tif]

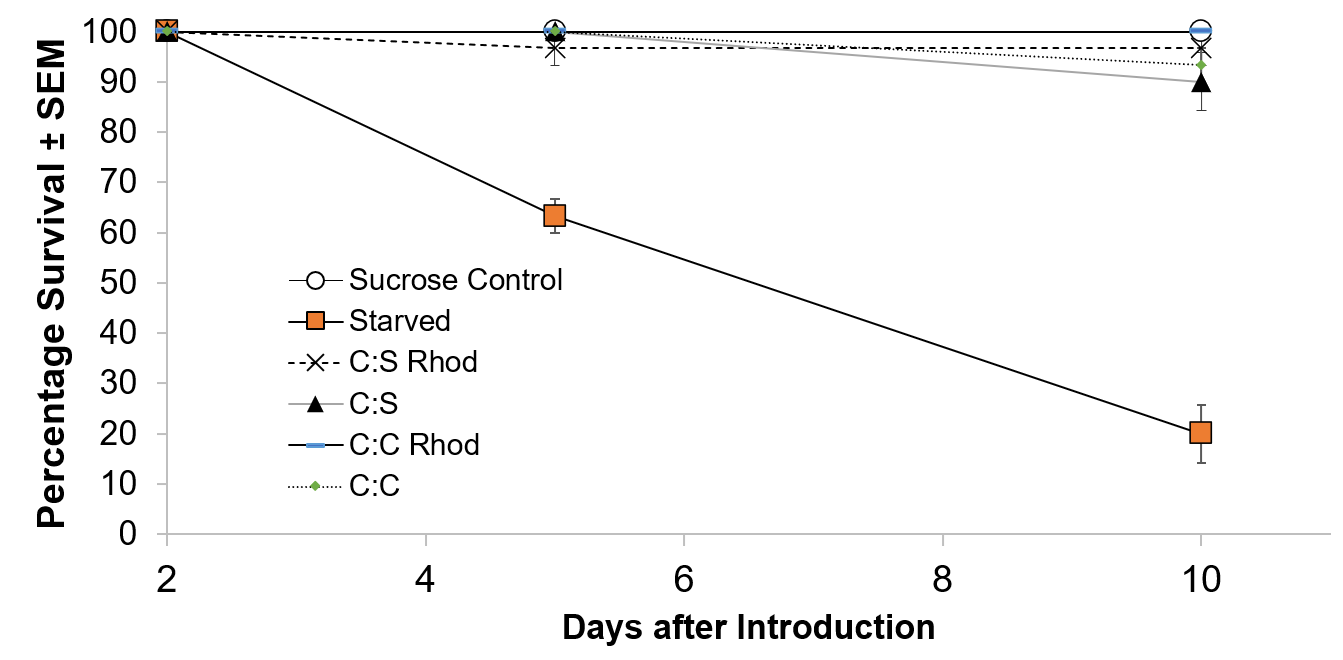

Supplement: S9 Fig — No differences in percentage mortalities were noted between the particle and Sucrose Control treatment throughout the entire experiment. The starved control produced statistically significant mortality compared to all other groups at day 5 and beyond. This indicates that mosquitoes feed on nanoparticle-sugar mixtures and they do not produce toxic effects at the concentrations utilized in this study. (TIF) [file pntd.0008365.s009.tif]

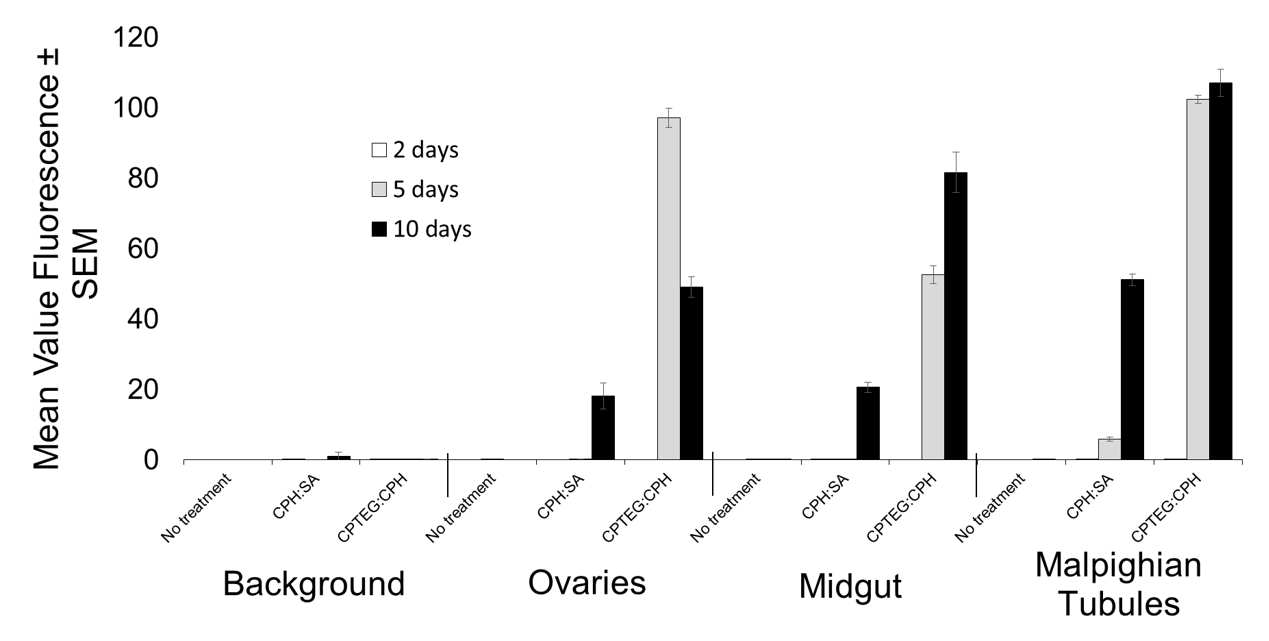

Supplement: S10 Fig — (TIF) [file pntd.0008365.s010.tif]
